# Identification of Key Sequence Motifs Essential for the Recognition of m^6^A Modification in RNA

**DOI:** 10.3390/biom16010097

**Published:** 2026-01-07

**Authors:** Aftab Mollah, Rushdhi Rauff, Sudeshi Abedeera, Chathurani Ekanayake, Chamali Thalagaha Mudiyanselage, Minhchau To, Helen Piontkivska, Sanjaya Abeysirigunawardena

**Affiliations:** 1 Department of Chemistry and Biochemistry, Kent State University, Kent, OH 44242, USA; 2 Department of Biological Sciences, Kent State University, Kent, OH 44242, USA; opiontki@kent.edu

**Keywords:** m^6^A modification, epitranscriptomic regulation, RNA–protein interactions, post-transcriptional RNA modifications, phage display

## Abstract

N6-methyladenosine (m^6^A) constitutes the most prevalent nucleotide modification within eukaryotic messenger RNA (mRNA). Variations in m^6^A levels are associated with numerous human diseases and health conditions, including various forms of cancer, diabetes, neurological disorders, male infertility, and obesity. Nevertheless, the molecular mechanisms underpinning the recognition of m^6^A by different ‘reader’ proteins remain incompletely elucidated. In this study, we used phage display to identify key sequence features that methyl readers recognize in m^6^A. This study shows that m^6^A modifications affect the mRNA interactome. A peptide motif recognizing m^6^A in DRACH sequences suggests a common recognition mechanism, though proteins may use different methods to detect m^6^A in less accessible areas. The sequence of the hnRNP A1 RRM domain that aligns with the newly discovered m^6^A-binding peptide, m1p1, is crucial for the binding of m^6^A-modified RNAs, indicating a strong link between the m1p1 sequence and m^6^A recognition, which is key for recognizing m^6^A-modified, unstructured RNAs. Gaining a comprehensive understanding of the evolutionary influence of m^6^A on its reader proteins may facilitate the identification of additional m^6^A readers. These signature peptides could enhance theranostic approaches across cancers, enabling more targeted therapies.

## 1. Introduction

**Figure 1:**
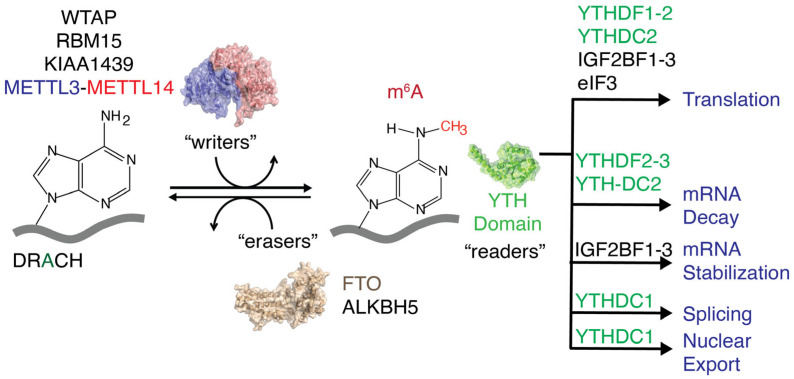
Methyltransferases (writers) and methylases (erasers) regulate cellular m^6^A levels. Different readers recognize m^6^A methylations and direct RNAs to various pathways.

N6-methyladenosine (m^6^A) is the most prevalent nucleotide modification found in eukaryotic mRNAs [1,2]. A methyl writer complex, comprising METTL3, METTL14, and Wilms’ tumor 1-associated protein (WTAP), that introduces methylations to adenines present within the DRACH (D = A, G, U; R = A, G; H = A, C, U) or RRACH sequence [1,3,4], a consensus sequence for the m^6^A writer complex and methyl eraser proteins, such as FTO and ALKBH5, removes m^6^A methylations. These m^6^A writers and erasers maintain a dynamic equilibrium of cellular m^6^A levels (Figure 1) and mediate various cellular processes during the epitranscriptomic regulation of gene expression [5,6,7,8]. Protein expression levels are altered at different developmental stages and in various human diseases, including cancer, diabetes, obesity, neurological disorders, and male infertility, by shifting the dynamic equilibrium of m^6^A methylations in either direction [9,10,11,12,13,14,15,16,17,18]. m^6^A reader proteins decode extracellular stress signals coded onto the transcriptome by m^6^A writers and erasers. These m^6^A reader proteins recognize methylations and guide RNAs toward different fates, such as degradation, splicing, increased translation, stabilization, and transport, with the help of their binding partners (Figure 1) [19,20,21,22,23,24].

Interestingly, m^6^A modifications occur in various RNA sequences and structural environments. The presence of m^6^A in 18 possible DRACH sequences and different secondary structural motifs requires unique reader proteins to detect these methylations [25]. Different recognition mechanisms can exist for reader proteins to specifically interact with m^6^A-modified RNA sequences and structures [26,27]. On the other hand, the binding mechanisms for different methylation sites are similar, but the affinity of the reader protein for m^6^A varies depending on its location [21,28]. In this context, the levels of reader protein expression will determine which m^6^A-modified RNAs are further processed [29]. Typically, non-covalent interactions such as ionic, hydrogen bonding, π–π, and hydrophobic interactions are formed between RNA and RBDs (RNA-binding domains of RNA-binding proteins) [22,30,31,32,33]. In contrast, unique short-range interactions are necessary to recognize these subtle modifications with high specificity and complementarity [34]. For example, YTH-domain-containing readers identify m^6^A-modified RNA through C-H ··· π interactions between the N6 methyl group and the aromatic side chains that form an aromatic cage within the YTH domain [12,35]. Furthermore, KH-domain-containing readers, such as IMP1, recognize m^6^A via a hydrophobic pocket present in the KH domain [31,36,37,38,39]. 

This study proposes a practical method for identifying the common features of m^6^A recognition mechanisms using phage display, a technique of directed evolution. Peptides selected through phage display will be examined for conserved sequence elements found within m^6^A-binding proteins identified via RNA pulldown assays. This research investigates the potential of peptide sequences enriched in phage display libraries as markers to detect m^6^A reader proteins.

## 2. Materials and Methods

### 2.1. RNA and DNA Oligonucleotide Preparations and Quantification

The chemically synthesized RNAs and DNAs used during this study were purchased from Horizon Discovery (Saint Louis, MO, USA) and Integrated DNA Technologies (Coralville, IA, USA), respectively. All chemically synthesized model RNA oligonucleotides were purchased from Horizon Discovery in HPLC-purified, 2′-ACE-protected form. The 2′-ACE-protected RNAs were deprotected by dissolving the RNAs in 2′-deprotection buffer (100 mM acetic acid, pH 3.8 adjusted with tetramethylenediamine, TEMED, Horizon Discovery) and incubating at 60 °C for 30 min. All the chemically synthesized model RNA oligonucleotides with a biotin conjugated at the 5′-end were dissolved in 2′-deprotection buffer and incubated at 60 °C for 2 h. Following incubation, the deprotected RNAs were dried completely using a vacuum concentrator and stored in a solid form at −20 °C in a freezer until further use.

The 2′-deprotected RNA was resuspended in TE buffer (10 mM Tris-HCl, pH 7.5, 1 mM EDTA, pH 8.0) to yield a 100 mM solution. The A_260_ values measured with the NanoDrop One (Thermo Scientific, Waltham, MA, USA) were divided by the appropriate extinction coefficient for each RNA to calculate the RNA solution concentration [35]. Aliquots of RNA working solutions were stored at −20 °C until future use. The chemically synthesized DNAs from IDT were resuspended in RNase-free water to a final concentration of 100 μM. Small aliquots of working solutions were stored at −20 °C for future use. Nucleotide sequences of all the RNA oligonucleotides and their respective extinction coefficients are listed in Table S1.

### 2.2. Phage Display

Structured RNAs used in phage display experiments (biotinylated MLR and MLR RNA) were folded by slow cooling to room temperature after heat denaturation at 95 °C. Multiple biopanning cycles (Figure S1) were performed in each phage display experiment as described previously [12]. Amplified phages from each biopanning cycle were used as the phage load in the subsequent biopanning cycle. A total of 4 biopanning cycles were performed with increasing stringency in the washing step (Table S2). In the second biopanning cycle, phages were counterselected against streptavidin-coated magnetic beads and plastic surfaces to eliminate off-target binding. In subsequent cycles, a random RNA and an RNA with a sequence present in the target RNA, located away from the m^6^A modification site, were used to eliminate nonspecific RNA-binding peptides and peptide binding to the target RNA at a site distant from the modification site, respectively. The eluted phages were titered at the end of the fourth biopanning cycle. Phage DNA from approximately 50 plaques was extracted, and the region with a randomized sequence was amplified using a standard method. The amplified PCR products were then purified using the E.Z.N.A Cycle Pure Kit (Omega Bio-Tek, Norcross, GA, USA). The size and purity of DNA fragments were tested using 1.5% agarose gel electrophoresis. PCR products of the correct size were sequenced (GenScript Biotech Corp, Piscataway, NJ, USA) to determine the unique dodecamer peptide fused to the pIII coat protein of each phage [12].

### 2.3. Site-Directed Mutagenesis, Overexpression, and Purification of hnRNP A1

Recombinant overexpression pET-24b(+) plasmid containing the hnRNP A1 coding sequence (p24bA1) was purchased from GenScript Biotech Corp (Piscataway, NJ, USA). Site-directed mutagenesis was performed on p24bA1 to generate a plasmid with hnRNP A1 RBD and three RBD mutants using Q5 site-directed mutagenesis kit (NEB). The presence of the desired mutations was confirmed by Sanger sequencing (Eurofins Genomics, Louisville, KY, USA). The hnRNP A1 protein, RNA-binding domain of hnRNP A1, and RNA-binding domain of hnRNP A1 with mutations were overexpressed using the recombinant overexpression plasmids (pET-24b(+)). Respective overexpression plasmids were then transformed into BL21(DE3) cells (NEB) using the manufacturer’s suggested protocol. Protein hnRNP A1 and its mutants were overexpressed in bacterial cells and purified using affinity chromatography (HisTrap FF column, Cytiva, Marlborough, MA, USA) as previously described [36]. Fractions with hnRNP A1 in high purity were combined and dialyzed against three buffer changes in TN Buffer (50 mM Tris-HCl, pH 7.5, 150 mM NaCl, 1 mM TCEP). Small aliquots of protein were stored at −80 °C after snap-freezing until use. Concentrations of the proteins were determined using measured A_280_ and respective molar extinction coefficients of hnRNP A1 and its mutants. The purity of purified proteins was confirmed by PAGE (Figure S2).

### 2.4. Mammalian Cell Culture

MDA-MB 231 cells were cultured in Dulbecco’s modified Eagle’s medium (DMEM) with 4.5 g/L of glucose (Corning, Corning, NY, USA), supplemented with 10% FBS (Gibco, Waltham, MA, USA) and 1% Penicillin/Streptomycin solution (Corning) [37]. They were incubated at 37 °C under 5% CO_2_. Cells were passaged upon reaching 70–80% confluency by trypsinization and split with a 1:5 dilution.

### 2.5. RNA Pulldown Assay

The MDA-MB-231 cells were cultured in three T75 flasks (Greiner Bio-One, Monroe, NC, USA) and lysed upon reaching confluence. The Pierce IP Lysis Buffer (Thermo Fisher Scientific, Waltham, MA, USA), supplemented with Halt Protease and Phosphatase Inhibitor Cocktail (Thermo Fisher Scientific) and SUPERase•In RNase Inhibitor (Invitrogen, Waltham, MA, USA), was used to lyse the cells according to the manufacturer’s protocol. Streptavidin-coated magnetic beads (NEB) were equilibrated with IP Lysis buffer (Thermo Fisher Scientific) and used for pre-clearing lysates by incubation at 4 °C for 20 min with shaking. Then, the lysates were incubated with tRNA (1.05 µg) and competitor RNA (540 pmol) for 20 min at 4 °C. The cell lysates were then equally divided into two for use with two different RNA targets (MR1 and UR1). These precleared cell lysates were then incubated with the target RNAs (270 pmol) for 1 h at 4 °C. The RNA–protein complexes were then incubated with streptavidin-coated magnetic beads (80 µL, NEB) pre-equilibrated in IP Lysis buffer. Several washes using TENT buffer (10 mM Tris-HCl, pH 8.0; 1 mM EDTA, pH 8.0; 250 mM NaCl; 0.3% (*v*/*v*) Triton X-100) and then with 100 mM Tris-HCl, pH 7.2, before elution of bound proteins by incubating at 95 °C for 10 min in elution buffer (400 mM Tris-HCl, pH 8.0) [38]. The eluted proteins were sent to the Lerner Research Institute’s Proteomics and Metabolomics Core (Cleveland Clinic, Cleveland, OH, USA) for LC-MS analysis for identification.

### 2.6. Calorimetric Titrations

Calorimetric titrations were conducted using a MicroCal PEAQ-ITC (Malvern Panalytical, Worcestershire, UK) for the HPLC-purified, purchased peptides (GenScript Inc., Piscataway, NJ, USA). Methylated and unmethylated target RNAs (20 µM) in Buffer F (50 mM Tris-HCl, pH 7.5, 150 mM NaCl) were titrated onto m1p1 peptide (1 µM) in Buffer F to determine the binding affinities of m1p1 peptide and its mutants to respective target RNAs, as described before [39,40]. To determine binding parameters for RNA–protein interactions, the RNA-binding domain of the hnRNP A1 protein and its mutants (50 µM) in Buffer F were titrated onto MR1 and UR1 RNAs (5 µM) in Buffer F. Equilibrium dissociation constants and thermodynamic parameters were obtained from the least-squares fitting of thermograms into a standard equation that assumed a single set of identical binding sites. All the titrations were performed in triplicate. Errors for measurements represent the standard deviations of the triplicates.

### 2.7. Bioinformatic Analysis

The RNA-binding proteins from the RNA pulldown assay were annotated using Gene Ontology (GO) Molecular Function terms using the Database for Annotation, Visualization and Integrated Discovery (DAVID Knowledgebase v2024q4) [41] and STRING (string-db.org) [42]. Alignments were carried out by using the ClustalW algorithm using MEGA 11 [43]. Peptide structure prediction was performed using an online structure prediction tool, PEPFOLD4 [44,45]. Pymol 3.1 was used for structural analysis [46].

### 2.8. Circular Dichroism

The Circular Dichroism (CD) spectra (190–320 nm) of m1p1 (10 μM) in a buffer (50 mM KCl, 20 mM sodium cacodylate, 0.5 mM EDTA, pH 7.6) were acquired using a JASCO J-810 spectropolarimeter connected to a Julabo F 25 water bath for temperature regulation. The average of three acquisitions was smoothed using the Savitzky–Golay algorithm (*n* = 13).

## 3. Results

Proteins bind to RNA and post-transcriptionally regulate processes such as translation, RNA splicing, transport, and decay [1]. Although nucleotide modifications are known to influence the binding affinities of proteins to RNA, the molecular mechanisms by which m^6^A and its neighboring sequences affect RNA–protein interactions are poorly understood. This study used phage display to explore how the m^6^A modification and adjacent nucleotides can exert selection pressure on proteins that bind to respective RNAs.

### 3.1. A Unique Peptide Sequence Was Enriched for m^6^A-Modified Single-Stranded RNAs

Two phage display experiments were conducted using two model RNAs, one with the most frequently methylated (GGACA) and the other with the least frequently methylated (AAACA) RRACH (R = A, G; H = A, C, U) sequence, which is the consensus sequence for the METTL3-METTL14 methyl reader complex. The exocyclic amine of the adenosine at the center of the RRACH sequence was methylated in MR1 and MR2 model RNAs, whereas UR1 and UR2 had the respective unmethylated sequences. A biotin tag was conjugated to the 5′-end of each model RNA to immobilize the RNA on streptavidin-coated beads. A randomized seven-nucleotide RNA sequence was added 5′ to the RRACH sequence to move the target sequence away from the streptavidin-coated beads that are used as the solid support in phage display and RNA pulldown experiments. Four biopanning cycles with increasing stringency were performed in each phage display experiment. After the fourth biopanning cycle, genomic DNA was extracted from individual phage colonies. Sanger sequencing was performed to determine the randomized 12-mer peptide sequence that is fused to the pIII coat protein of each enriched phage.

**Figure 2:**
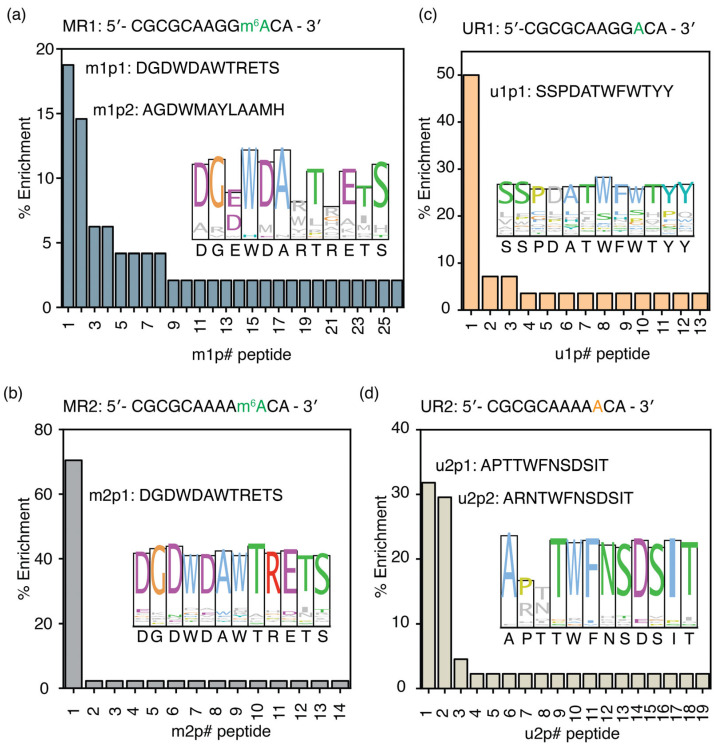
Unique peptide sequences enriched in phage displays for m^6^A-modified RNAs. The percentage enrichment of peptides obtained from phage display experiments performed with (**a**) MR1 (5′-CGCGCAAGGm^6^ACA-3′), (**b**) UR1 (5′-CGCGCAAGGACA-3′), (**c**) MR2 (5′-CGCGCAAAAm^6^ACA-3′), and (**d**) UR2 (5′-CGCGCAAAAACA-3′) model RNAs is shown. Sequence logos, drawn in Clustal colors, for all enriched peptides for each target RNA are shown in the inset. The # represents the peptide number.

The phage display experiment performed using MR1 (5′-CGCGCAAGGm^6^ACA-3′), a methylated RNA, as the target produced 26 unique peptide sequences. The percentage of enrichment for m1p1 (DGDWDAWTRETS), the most enriched peptide obtained for MR1, is 18.8%, whereas the second most enriched peptide, m1p2 (AGDWMAYLAAMH), has a percentage occurrence of 14.6%. Interestingly, the two most enriched peptides, m1p1 (DGDWDAWTRETS) and m1p2 (AGDWMAYLAAMH), exhibit significant sequence similarity (Figure 2a and Table S4), particularly at positions 4 and 7, which contain two aromatic amino acid residues. The consensus sequence obtained from the ClustalW alignment of all the enriched peptides (DGEWDARTRETS) is similar to the two most enriched peptides. However, the aromatic amino acid found at position 7 in the two most enriched peptides is replaced by a positively charged amino acid in the consensus peptide sequence. In addition to the most methylated RRACH sequence, two more model RNAs with the least methylated RRACH sequence (MR2) and a random sequence (MR3) with an m^6^A were used as targets in two separate phage display experiments. The most enriched peptide selected for the MR2 model RNA with the AAm^6^ACA sequence, m2p1, was found to be the same peptide (m1p1: DGDWDAWTRETS) selected for MR1 RNA (Figure 2b and Table S4). The entire m1p1 sequence was conserved among peptides targeting MR2 (Figure 2b, inset). Interestingly, the m1p1 peptide has a higher percentage enrichment (70.5% enrichment) for the least abundant RRACH sequence, AAm^6^ACA, compared to the GGm^6^ACA sequence (18.8%).

The m1p1 peptide sequence preferably binds to the two RNAs containing the RRACH sequence. However, this peptide may recognize the RRACH sequence but not the m^6^A methylation associated with it. Phage display experiments using unmethylated versions of the MR1 and MR2 RNAs were performed to determine whether the peptide selected for MR1 and MR2 could recognize the RRACH sequence without methylation. The most enriched peptide (u1p1: SSPDATWFWTYY) for UR1 target RNA had a 50.0% enrichment (Figure 2c and Table S5). The u1p1 sequence is also the consensus sequence for all enriched peptides for the UR1 target, due to its very high abundance in the enriched peptide pool (Figure 2c, inset). Among all the peptides selected for unmethylated target RNA, UR2, u2p1 (APTTWFNSDSIT), and u2p2 (ARNTWFNSDSIT) peptides have 31.8% and 29.5% enrichment, respectively (Figure 2d and Table S6). Although both methylated target RNAs enriched the same peptide, peptide sequences that bind to unmethylated RRACH sequences have contrasting sequences. The amino acid sequences of the peptides that bind to the UR2 model RNA are conserved except for the amino acids at positions 2 and 3 (Figure 2d, inset). Most importantly, the most enriched peptides selected for the unmodified RNA target (UR2) had no sequence similarity to those selected for its modified counterpart (MR2). These observations confirm that the m1p and m2p peptide pools recognize methylations within the RRACH sequence; however, they do not recognize the RRACH sequence.

Another phage display experiment was performed to investigate whether the RRACH sequence influences the mode of m^6^A recognition by proteins. A target RNA with a non-RRACH sequence with an m^6^A modification was used for this phage display. The most enriched peptide from phage display of a random m^6^A-modified RNA sequence, m3p1 (AGDWESWTNGSW), shares sequence features with m1p1, m2p1, and m1p2 (Figure S3). This observation indicates the presence of a signature sequence motif that can recognize m^6^A methylations. However, changes in the percentage enrichment of the preferred peptide, m1p1 (DGDWDAWTRETS), across different RRACH sequences suggest that although methylated RRACH sequences preferentially bind to unique peptide sequences, their affinities for different RNAs vary with the target RNA sequence.

### 3.2. Enriched Peptides Can Be Classified into Clusters with Similar Physicochemical Properties

**Figure 3:**
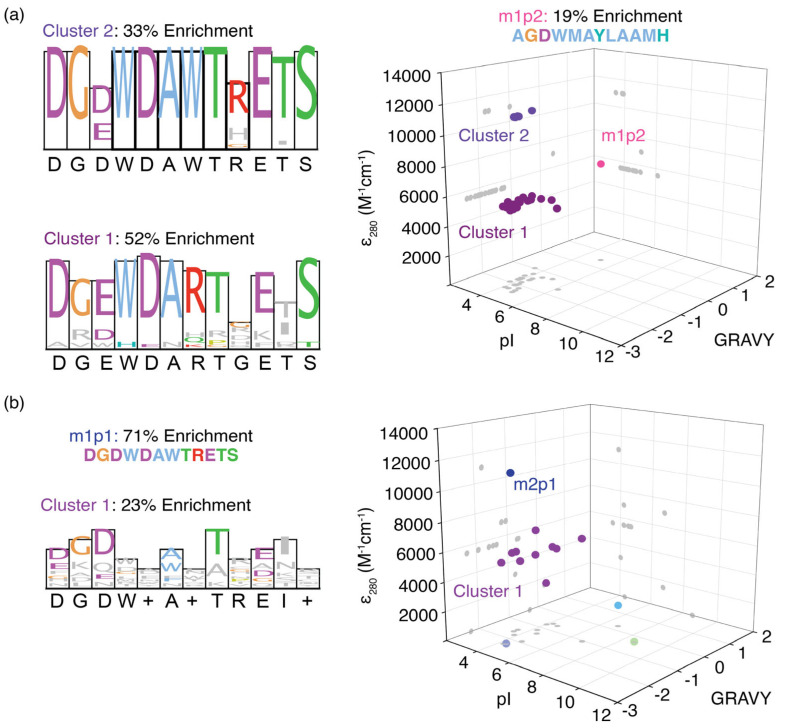
Separation of enriched peptides for (**a**) MR1 and (**b**) MR2 methylated model RNAs based on their grand average hydropathicity (GRAVY), pI, and molar extinction coefficient at 280 nm (*ε*280). Each spear represents a single peptide enriched in phage display, and they are projected in three axial planes (gray dots). Three and two distinct peptide clusters are observed for those enriched in phage displays for MR1 and MR2, respectively. Sequence logos for each cluster are also shown next to the respective 3D plot.

Based on their physicochemical properties, three distinct peptide sequence clusters were identified when pooling m1p peptides (Figure 3a). The first cluster had a consensus sequence similar to the most enriched peptide, m1p1, whereas the m1p2 peptide showed contrasting physicochemical properties and formed a unique cluster. The third cluster of peptides had a consensus sequence (DGDWDARTGETS) similar to that of the entire peptide pool (DGDWDARTRETS). In addition, all the peptides enriched for the MR2 model RNA, except for m1p1 (m2p1: DGDWDAWTRETS), had less than 5% enrichment individually. These peptides clustered together in the physicochemical analysis (Figure 3b). This cluster of peptide sequences showed a similar consensus (DGDW+A+TREI+) to that of the m2p1 peptide sequence (DGDWDAWTRETS), with few exceptions. These observations suggest that unique peptide sequences recognize m^6^A modifications exclusively found within RRACH sequences.

The physicochemical properties of the enriched peptide sequences in each phage display for MR1 and MR2 were analyzed to understand the mechanism of m^6^A recognition by these peptides. The average pI of the m1p and m2p peptide pools is 4.3 and 4.2, respectively, indicating that peptides in both pools carry an overall negative charge at physiological pH (Tables S3 and S4). The most enriched peptide for MR1 and MR2 target RNAs (m1p1) has only one positively charged amino acid; nevertheless, three negatively charged aspartic acid residues are present at positions 1, 3, and 5 in the dodecamer peptide sequence. These negatively charged amino acid residues are highly conserved among all enriched peptides. The presence of aspartate and glutamate residues is rarely observed in RNA-binding proteins. However, aspartate and glutamate side chains can form hydrogen bonds through the Watson–Crick edge of guanosines. On the other hand, positively charged amino acids in RNA-binding proteins often form ionic interactions with the negatively charged phosphate backbone. However, ionic interactions between the RNA backbone and positively charged amino acid side chains are not sequence specific. RNA methylations are frequently recognized by excluding water from the methylation site through hydrophobic amino acid side chains. The grand average of hydropathicity (GRAVY), which measures the hydrophobic nature of any given peptide, is found to be at an average of −1.45 and −1.59 for m1p and m2p peptide pools, respectively, suggesting an overall hydrophilic nature for the selected peptides (Tables S3 and S4). Most dodecamer peptides enriched in phage displays for m^6^A-modified RNA targets feature aromatic amino acids at positions 4 and 7. The tryptophan residues at position 4 (W4) of the most enriched peptide, m1p1, are highly conserved (Figure 2a, inset) among all enriched peptide sequences. Therefore, the presence of aromatic amino acids at positions 4 and 7 of both m1p1 and m1p2 peptides suggests that these aromatic amino acids play a role in binding to methylated RNAs.

### 3.3. The Location of m^6^A on Structured RNA Influences Its Recognition by Proteins

**Figure 4:**
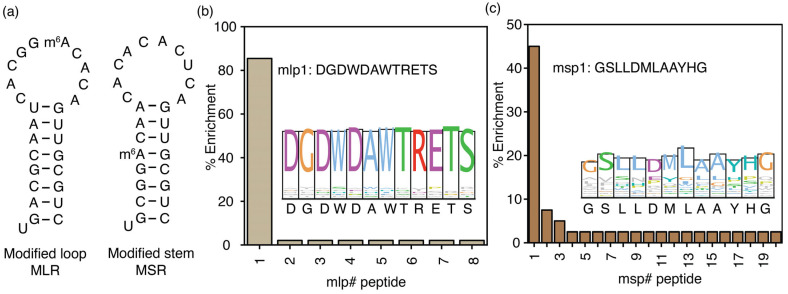
The location of m^6^A in structured RNAs influences the selection of peptide binding partners. (**a**) Two hairpin RNAs containing m^6^A nucleotide modifications in the loop (MLR) and stem (MSR) utilized in phage display are presented. The percentage of peptide enrichment from the phage display experiments using (**b**) MLR and (**c**) MSR model RNAs is shown with sequence logos in the respective insets. The # represents the peptide number.

RNA modifications, including m^6^A modifications, are observed in both unstructured and structured regions of RNA transcripts [47,48]. This study also hypothesizes that mechanisms of m^6^A recognition and the m^6^A interactome can vary depending on structure. Phage display experiments used two model RNAs with hairpin structures to test this hypothesis. In the MLR model RNA, the RRm^6^ACH sequence (GGm^6^ACA) was added to the hairpin loop. In contrast, in the MSR model RNA, the hairpin stem comprised the RRm^6^ACH sequence (Figure 4a). A control RNA containing the same stem as the MLR model RNA was used in counter-selection during the fourth biopanning cycle of MLR RNA to ensure the enriched peptides bind specifically to the methylation site in the loop. Similarly, during the fourth biopanning cycle for the MSR model RNA, enriched phages were counterselected against a hairpin RNA without the DRACH sequence in the stem (Table S7). Neither of these control RNAs had any m^6^A modifications incorporated into RRACH sequences. The m1p1 peptide (DGDWDAWTRETS), enriched in phage display experiments using the two single-stranded methylated target RNAs, was found to be the most enriched peptide (85.4%) selected for the MLR model RNA, the hairpin RNA with the methylation in the hairpin loop (Figure 4b). The entire m1p1 sequence is conserved among the selected peptides for MLR RNA (Figure 4b, inset). Phage display was performed with the model RNA with the DRACH sequence in the stem of the RNA hairpin as the target; the msp1 peptide with the GSLLDMLAAYHG peptide sequence is enriched (Figure 4c), in contrast to the DGDWDAWTRETS peptide sequence, which shows the highest enrichment (45.0%) for all the other DRACH sequence-containing model RNAs, including the hairpin RNA with the DRACH sequence in the hairpin loop. The msp1 peptide illustrates distinct properties compared to the DGDWDAWTRETS sequence (Figure 4c). The average GRAVY value for the enriched peptides for the MSR model RNA is 0.25, which suggests the hydrophobic nature of this peptide pool. The most enriched peptide, msp1, is more hydrophobic (GRAVY = 0.608; Table S7) than the mlp1 (DGDWDAWTRETS) peptide (GRAVY = −1.76; Table S8). This observation indicates different mechanisms for recognizing methylations in stem or double-stranded regions compared to those in single-stranded or loop regions of the RNAs.

### 3.4. Affinity of Enriched Peptide to m^6^A-Modified RNAs Varies with RNA Structure and Sequence

**Figure 5:**
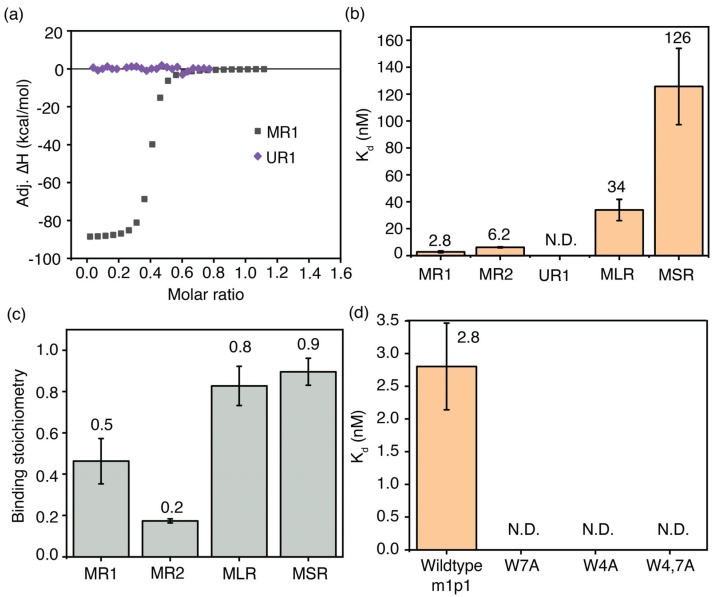
The most enriched peptide, m1p1, in phage display for m^6^A–modified RNAs tightly binds its target RNA. (**a**) Representative ITC thermograms for the titrations of MR1 (gray square) and UR1 (purple diamonds) model RNAs onto m1p1 peptide at 37 °C; (**b**) equilibrium dissociation constants; and (**c**) binding stoichiometry for the m1p1-RNA complexes are shown. (**d**) Equilibrium dissociation constants for the MR1 model RNA complexed with m1p1 peptide and its single mutants (W7A, W4A) and the double mutant (W4,7A) are shown.

The percentage enrichment of peptides after stringent washes is an indirect measure of each peptide’s binding affinity. The binding affinity of m1p1 towards the m^6^A-modified target RNA (MR1) was compared with that of its unmodified target RNA counterpart (UR1) to confirm the relationship between phage selection and binding affinity. Isothermal calorimetry (ITC) experiments were performed using a MicroCal PEAQ-ITC calorimeter. In these experiments, the respective RNA in TK buffer (50 mM Tris-HCl, pH 7.5, and 150 mM KCl) was titrated into 1 µM peptide in the same buffer. The heat released upon adding MR1 to m1p1 indicates an interaction between the two (Figure 5a). The heat generated during titration was plotted against the molar ratio of RNA to peptide. These binding curves were fitted to a binding model that assumes a single set of identical sites. The MR1-m1p1 complex had a K_d_ of 2.8 ± 0.7 nM (Figure 5b). Calorimetric titrations with unmethylated UR1 RNA and m1p1 showed no significant heat change, even at millimolar concentrations of the titrant, indicating a much lower binding affinity compared to m^6^A-modified RNA (Figure 5b). These calorimetric data suggest that the m1p1 peptide, which is selectively enriched during phage display experiments, binds specifically to loosely structured m^6^A-modified RNAs.

Phage display experiments for several m^6^A-modified model RNAs bind to m1p1 peptide and other peptides similar in sequence to m1p1. However, contrasting percentage enrichment was observed for m1p1 for different m^6^A-modified model RNAs, suggesting a change in binding preference for each model RNA. ITC measurements were performed to confirm that the percentage enrichment of the peptide correlates with the binding affinity. The two single-stranded model RNAs with the highest percentage enrichment of the m1p1 peptide, MR1 (2.8 ± 0.7 nM) and MR2 (6.2 ± 0.3 nM), illustrated the tightest affinity to the m1p1 peptide (Figure 5b and Table S7) among RNAs tested. In contrast, the two hairpin model RNAs, MLR and MSR, had a lower binding affinity to m1p1. The MLR model RNA with the RRm^6^ACH sequence on the loop had a higher affinity (34 ± 8 nM) than the MSR model RNA (125 ± 28 nM), in which the RRm^6^ACH sequence is placed on the stem (Figure 5b). Although a peptide (msp14: AGDWESWTNGSW) similar in sequence to m1p1 was enriched (2.5% enrichment), the sequence of the most enriched peptide (msp1: GSLLDMLAAYHG) was significantly different from m1p1, resulting in a lower binding affinity of MSR to the m1p1 peptide. Interestingly, a 1:2 binding stoichiometry was observed for MR1 and the m1p1 peptide, suggesting that multiple copies of the peptide participate in methyl recognition (Figure 5c). Perhaps due to steric reasons, approximately 1:1 binding was observed for MLR and the m1p1 (mlp1) peptide (Figure 5c).

### 3.5. Aromatic Amino Acids Sidechains in m1p1 Peptide Are Essential for m^6^A Recognition

The YTH-domain-containing proteins recognize N6 methylation by positioning the methyl group in an aromatic cage formed by tryptophan and phenylalanine side chains [22,49] in which hydrogens of the methyl group form C-H---π interactions with the aromatic cage [50]. The PEPFOLD4 algorithm predicts an α-helical secondary structure for the m1p1 peptide, which was confirmed using circular dichroism (CD) spectroscopy (Figure S4). In this α-helix, W4 and W7 of the peptide are close, and their sidechain aromatic indole rings point in the same direction. The ability of these two tryptophan residues of m1p1 to recognize the m^6^A methyl group was tested using ITC. In these calorimetric measurements, MR1 was titrated onto three mutant m1p1 peptides, where W4 and W7 were substituted by alanine residues, respectively. A double mutant was also tested, where both W4 and W7 were mutated to alanine. All three mutant peptides did not bind significantly to the MR1 model RNA (Figure 5d and Figure S5 and Table S9), suggesting that both tryptophan residues play an essential role in recognizing m^6^A-containing RNAs. The need for aromatic amino acids in m1p1 to recognize m^6^A-modified RNA further clarifies the role of aromatic cages in YTH-domain-containing proteins in identifying methylated RNAs.

### 3.6. Several Proteins with RRM and KH Domains Bind to Single-Stranded m^6^A RNA

The m1p1 peptide can serve as a marker for identifying novel m^6^A-binding proteins, as it recognizes N6-methylations across diverse sequences and structural contexts. Protein BLAST (pBLAST 2.16.0) searches were performed to find eukaryotic proteins with sequences similar to that of the m1p1 peptide. The Expect values (E-values) for all protein hits were high, likely due to the short query length, which may have decreased the reliability of the results. Additionally, none of the known m^6^A reader proteins appeared among the hits from these pBLAST searches. As a result, multiple sequence alignments were performed for m1p1 and established m^6^A reader proteins with YTH domains (YTHDF1/2/3, YTHDC1/2) to evaluate potential sequence similarity. Although a significant sequence alignment was observed between the m1p1 peptide and YTH domains, the aligned YTH sequences did not match the methylation recognition site, possibly because the abundance of aromatic amino acid side chains clustered together created multiple sites for good sequence alignment (Figure S6).

**Figure 6:**
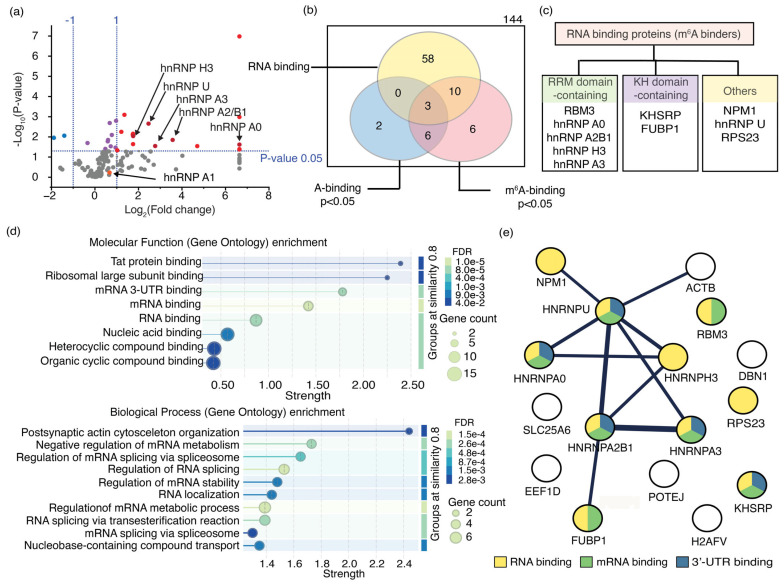
The most enriched proteins that bind to m^6^A-modified RNAs are identified using an RNA pulldown assay. (**a**) A volcano plot shows the relative enrichment of proteins in RNA pulldown assays for m^6^A-modified RNA compared to its unmodified counterpart, with statistical significance indicated. The vertical dashed lines indicate the 2-fold enrichment for either RNA, whereas the horizontal dashed line represents the threshold for the statistically significant binding events (*p* = 0.05). Proteins that bind preferably to m^6^A-modified and unmodified RNAs, with statistical significance, are shown in red and blue circles, respectively. Purple circles represent the protein that has no preference for either RNA. hnRNP proteins are shown in dark red; (**b**) The Venn diagram shows the number of RNA-binding proteins that bind to either RNA with statistical significance (*p* < 0.05, *n* = 3); (**c**) Different types of RNA-binding motifs of enriched proteins; (**d**) Molecular functions and biological processes in which m^6^A-binding proteins are involved are shown; (**e**) STRING analysis illustrates physical interactions of m^6^A-binding proteins enriched in the pulldown assay.

Primarily due to limitations in using short amino acid sequences for pBLAST searches, an RNA pulldown assay was performed to selectively enrich proteins that bind m^6^A-modified RNAs more favorably than their unmodified counterparts. Proteins enriched in the pulldown assay exhibited a higher affinity for methylated RNAs. These RNA pulldown assays utilized the same single-stranded RNAs used in the phage display as baits. Proteins extracted from MDA-MB-231 cell lysates were precleared with streptavidin-coated magnetic beads (NEB, USA) and control RNAs before incubating with m^6^A-containing RNAs and their respective unmethylated counterparts used as bait. Multiple rounds of washing with increasingly stringent buffers were performed to ensure the selection of tight binders—pulldown assays for methylated and unmethylated single-stranded RNA baits enriched 144 proteins (Figure 6a,b). The enrichment was statistically significant (*p*-values ≤ 0.05; *n* = 3) for only 27 of these 144 proteins. Sixteen proteins in this group of 27 showed a greater than two-fold preference (M/U > 2) for the methylated RNA target (MR1) compared to its unmethylated counterpart (UR1) (Figure 6b and Table S10). Two proteins displayed more than a two-fold preference (M/U < 0.5) for the unmethylated RNA (Figure 6b). Nine proteins showed no preference (0.5 < M/U < 2) between methylated and unmethylated RNAs (Figure 6b).

The GO enrichment analysis revealed that only 10 of the 16 significantly enriched proteins with a higher preference for MR1 are well-characterized RNA-binding proteins. Seven of these 10 RNA-binding proteins interact with mRNA; five specifically bind to their 3′-UTR (Figure 6d). Additionally, the GO enrichment analysis indicated that all 10 RNA-binding proteins enriched in the assay are localized in the nucleus (Figure S7), five of which are hnRNP proteins associated with the spliceosome complex (Figure 6d). Notably, several hnRNP proteins, such as hnRNP A2B1, hnRNP C, and hnRNP G, are known to recognize m^6^A methylations in the nucleus and participate in RNA splicing. These findings imply that hnRNP proteins enriched in the RNA pulldown assay likely function as m^6^A readers. For example, hnRNP A2B1 demonstrated a 12-fold increase in affinity for methylated RNA (MR1) compared to its unmethylated counterpart (UR1), establishing it as an m^6^A reader protein (Table S10). Furthermore, hnRNP C, another established m^6^A reader protein, exhibited a 2.4-fold greater preference for MR1 than UR1. Unfortunately, hnRNP C was excluded from further analysis due to low statistical significance (*p*-value > 0.05). The presence of these two known m^6^A readers, both showing more than a 2-fold higher binding preference for methylated targets, further supports the potential of this RNA pulldown assay to identify a novel m^6^A-binding protein. STRING analysis indicates that the enriched hnRNP proteins can form functional complexes (Figure 6e). Furthermore, the proteins NPM1, FUBP1, and ß-actin, which were enriched in the pulldown assay, form complexes with hnRNP proteins. These proteins are likely to bind to methylated RNAs either independently or together, thereby strengthening their roles in cellular processes. Among the enriched proteins, those not known to bind RNA do not interact with hnRNP proteins, except for ß-actin, which suggests a probable interaction with m^6^A methylation indirectly through the hnRNP U protein. Among the 144 proteins enriched in the RNA pulldown assay, 12 hnRNP proteins showed a binding preference for MR1 (Figure 6a). All hnRNP proteins that significantly bind to m^6^A-modified RNA, except for hnRNP U, have at least one RNA recognition motif (RRM), a protein domain that interacts with single-stranded RNAs (Figure 6c). RRMs are often found in proteins involved in RNA processing, RNA stability, and transport. Strong sequence conservation was observed for RRMs in hnRNP proteins. Multiple sequence alignments were performed to identify sequence motifs in these enriched hnRNP proteins that enable m^6^A recognition.

### 3.7. Enriched Proteins Contain Sequences That Are Similar to Those of the m1p1 Peptide

**Figure 7:**
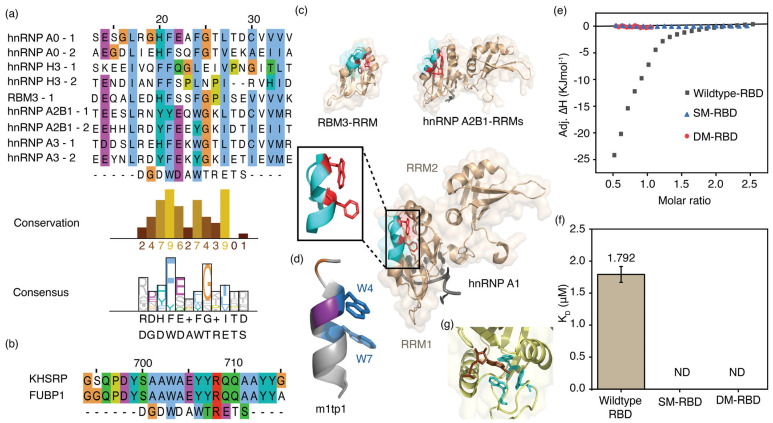
Sequences of RRM and KH domains of enriched proteins align with the m1tp1 sequence. Multiple sequence alignments of (**a**) RRM and (**b**) KH domains in enriched proteins from RNA pulldown assays with the m1tp1 peptide are shown. Sequence conservation and consensus for RRM domains are also displayed; (**c**) The three-dimensional structures of the RNA recognition motifs (RRMs) of RBM3 (PDB ID: 7eb1), hnRNP A2B1 (PDB ID: 5ho4), and the two RRMs of hnRNP A1 (RRM1 and RRM2; PDB ID: 8x0n). The inset shows the hnRNP A1 RBD structure portion that sequentially aligns with m1tp1; (**d**) The structure of m1tp1 predicted by the PEPFOLD4 algorithm; (**e**) Thermograms for titrations of MR1 onto wild-type and mutant hnRNP A1 RBDs; (**f**) Equilibrium dissociation constants for MR1 complexes with wild-type and mutant hnRNP A1 RBDs. The average of triplicate measurements for each titration is shown. Error bars represent the standard error from three independent measurements; (**g**) The X-ray crystal structure of the YTHDC1 YTH-domain complexed with m^6^A nucleotide (PDB ID: 6zda).

A multiple sequence alignment of the 9 RRMs from 5 proteins, performed with the ClustalW algorithm, showed good alignment within their RNA-binding domains (Figure S8). The sequence of the m1p1 peptide also aligns well with the selected RRMs (Figure 7a). Similarly, a good sequence alignment was observed between the m1p1 peptide and protein sequences of enriched KH-domain-containing proteins adjacent to the KH-domain. Interestingly, investigation of the X-ray crystal structures of RRMs from the enriched proteins (Figure 7c) shows that the regions of the RRMs that aligned with m1p1 are structurally similar to the predicted structure of m1p1 (Figure 7d).

The hnRNP A1 protein was chosen for further testing of its ability to bind m^6^A-modified RNA due to its well-characterized structure and RNA-binding properties. Isothermal calorimetric titrations were carried out using the RBD of hnRNP A1 and various RNAs. The RBD of hnRNP A1 protein (50 µM) in TK buffer was titrated onto MR1 and UR1 (both 5 µM) in the same buffer. Thermograms were then fitted to a binding model assuming a single set of identical sites. The hnRNP A1 RBD and MR1 complex had a K_d_ value of 1.8 ± 0.1 µM (Figure 7e,f). The 1:2 binding stoichiometry (*n* = 0.48) for hnRNP A1 RBD and methylated RNA can be explained by the presence of two RRMs, each with an FEQ(W/Y) sequence aligned with the m1p1 sequence. Surprisingly, similar calorimetric titrations between UR1 and the hnRNP A1 RBD did not release significant binding heat, indicating weak or no binding over the concentration range tested (Figure S9). The ability of hnRNP A1 RBD to bind more strongly to MR1 than to UR1 confirms its function as an m^6^A-binding protein.

### 3.8. Aromatic Amino Acid Sidechains in the Aligned Region of hnRNP A1 Are Essential for Binding to m^6^A-Modified RNAs

The multiple sequence alignment of m1p1 and hnRNP A proteins using the ClustalW algorithm showed that the two tryptophan residues (W4 and W7) of the m1p1 peptide closely align with two aromatic amino acid residues of hnRNP A1 RRMs (Figure 7a). Additionally, aspartic acid and threonine residues at positions 5 and 11 align well with two glutamic acid and threonine amino acid residues of the hnRNP A1 protein. Compared to the m1p1 peptide, the region of hnRNP A1 that aligns with it shows the same secondary structure. Besides the similarity in secondary structure between the m1p1 peptide and the corresponding part of the protein, a notable resemblance was also seen in the orientation of the two tryptophan (W4 and W7) side chains in m1p1 and the two aromatic side chains, F34 and W37, in the aligned sequence of hnRNP A1. These two aromatic amino acid side chains in the m1p1 peptide are essential for binding to methylated RNAs (Figure 5d and Figure S5). Therefore, F34 and W37 of hnRNP A1, which align with the aromatic residues in m1p1, may also contribute to m^6^A recognition. Calorimetric titrations were performed using the F34A and W37A single mutants of hnRNP A1 and the respective double mutants. The hnRNP A1 mutants (50 µM) in TK buffer were titrated against MR1 (5 µM) in TK buffer. None of the mutants showed binding to methylated RNA (Figure 7e,f and Table S11), indicating that F34 and W37 of hnRNP A1 are necessary for recognizing m^6^A-modified RNA. These findings suggest that the hnRNP A1 protein can recognize m^6^A methylations and that the aromatic amino acid side chains (F34 and W37) are crucial for m^6^A recognition. All these observations suggest that proteins with sequence features similar to m1p1 can recognize m^6^A modifications in single-stranded, unstructured regions.

## 4. Discussion

The influence of m^6^A modification on evolutionary conservation has been examined across diverse organisms and within the human population [51,52]. However, the molecular mechanisms by which dynamic alterations in m^6^A induce phenotypic changes in organisms remain inadequately explored. The addition of m^6^A modifies the mRNA interactome and determines the fate of the corresponding mRNA, thereby impacting the proteome [53,54,55]. This research aimed to elucidate the molecular mechanisms underlying the sequence- and structure-dependent recognition of m^6^A. In phage display experiments, sequences of peptides enriched for all methylated model RNAs significantly differed from those enriched for unmodified model RNA counterparts, strongly indicating the capacity of N6-methyl groups to influence protein binding partners. The m^6^A modification appears to exert distinct selection pressure on its binding partners [24,56]. This stringent selection is available for various DRACH sequences, including those within secondary structures, and for highly accessible m^6^A modifications found in single-stranded RNAs.

Interestingly, peptide sequences enriched through phage display targeting the most prevalent m^6^A-modified RRACH sequence appear to exhibit notable similarity. Although these enriched peptide sequences can be categorized into three clusters based on their physicochemical properties, the consensus sequences of the two clusters with the highest enrichment levels differ significantly by only two amino acids at positions 7 and 9 (Figure 2a,b). This finding indicates a highly stringent selection process for m^6^A-binding peptide sequences in these phage display experiments. The most enriched peptide sequence contained merely one positively charged amino acid, suggesting that the interaction is unlikely to be a non-sequence-specific electrostatic attraction between a positively charged side chain and the phosphate backbone. The initial rationale for the presence of negatively charged aspartates and glutamates was their potential to form hydrogen bonds with nucleotide bases via Watson–Crick or Hoogsteen edge interactions. Similarly, serine and threonine residues, which are abundant in the enriched peptides, may also facilitate hydrogen bonding with nucleotide bases. These observations imply a probable sequence-dependent mechanism of N6 methylation recognition. Moreover, four RNA sequences with m^6^A modifications tested in this study (MR1, MR2, MRR, and MLR) showed enrichment for similar peptide sequences, indicating a common recognition mechanism for various single-stranded m^6^A sites. The ability of these RNAs to bind the m1p1 peptide with nanomolar affinity confirms that this peptide recognizes methylations in diverse RNA structures and sequences. The presence of two tryptophan residues at positions 4 and 7 is a common feature among many enriched peptides for all three single-stranded targets examined and the hairpin RNA with m^6^A in the loop. Interestingly, only the hairpin RNA with m^6^A in its stem region showed enrichment of peptide sequences lacking these two conserved tryptophan residues. Notably, many known m^6^A readers, especially YTH-domain-containing proteins such as YTHDC1 and YTHDF1, utilize a cavity formed by aromatic amino acid side chains to recognize m^6^A [22,49]. Structural predictions of the m1p1 peptide indicate that the two tryptophan residues are positioned in a manner that could generate an aromatic cage capable of interacting with N6 methylations. Calorimetric analyses of the peptide-RNA interactions suggest that multiple peptides are required for effective recognition of the methylation, supporting a model involving an aromatic cage constituted by multiple m1p1 units. This evidence collectively supports a recognition mechanism for m^6^A that involves an aromatic cage formed by several peptides. The existence of a universal recognition mechanism across different sequences and structural contexts minimizes the necessity for a distinct set of reader proteins. In scenarios where a single reader protein interacts with methylations across various unstructured RNAs, writer and eraser enzymes predominantly regulate gene expression [57,58]. In such occasions, alterations in the expression levels of writer and eraser proteins can selectively methylate or demethylate distinct RRACH sites, thereby regulating the fate of corresponding mRNAs [58]. Although reader proteins employ a universal mechanism to recognize m^6^A modifications across diverse sequence and structural contexts, the affinity of a given reader can vary, with m^6^A reader expression levels determining target selectivity [1]. Additionally, neighboring amino acid residues can help modulate the affinity of aromatic cages for m^6^A, thereby influencing RNA-binding partner selection among different reader proteins [22].

Although target RNAs containing m^6^A within unstructured or flexible regions favor the m1p1 peptide sequence, the MSR hairpin RNA, in which the m^6^A is situated in the hairpin stem, exhibits a preference for a peptide with contrasting sequence characteristics and physicochemical properties. This observation suggests the existence of complementary mechanisms for recognizing m^6^A methylations across different secondary structural contexts. N6-methylations within double helical regions are exposed in the major groove; consequently, any protein domain responsible for detecting N6-methylations in helical regions should have unobstructed access to the major groove. The adjacent hydrophobic side chains of the reader protein can form pockets that encapsulate the methyl group and displace water molecules from the primary hydration layer of the RNA [59].

Interestingly, the signature sequences of m^6^A-binding peptides were observed in proteins enriched in RNA pulldown assays performed using m^6^A-modified RNAs. Since phage display experiments and RNA pulldown assays are performed with the same target RNAs, any discrepancies arising from sequence-dependent m^6^A recognition were eliminated. RNA pulldown assays have enriched proteins containing RRM and KH domains. Both RNA-binding domains are found in proteins that regulate transcription and translation. The strong alignment of the m1p1 peptide sequence with sequences of RRM- and KH-domain-containing proteins suggests that these domains can recognize methylations within unstructured RNA regions. Five out of ten RNA-binding proteins enriched in the RNA pulldown assay contained RRM domains. Among the five proteins enriched with RRM domains, four belong to the hnRNP family. While some hnRNPs, such as hnRNP A2B1, hnRNP C, and hnRNP G, are recognized as m^6^A readers, it is noteworthy that none of the enriched hnRNPs except hnRNPA2B1 have been previously identified as m^6^A-binding proteins. Interestingly, hnRNP A1/B2 has been observed to bind to m^6^A-modified miRNAs and inhibit the loading of those miRNAs to the RISC complex. The m^6^A-modified miRNAs are consequently loaded into vesicles for extracellular transport. It is likely that hnRNP A1/B2 interacts with m^6^A nucleotide modification using an aromatic cage in their RRM domain, as proposed in this study [59]. Although the m^6^A binding properties of hnRNP A1 have not been reported previously, its ability to bind to m^6^A-rich RNAs such as SARS-CoV-2 RNA and influence biological processes is well known [60]. Sequence alignment of hnRNP A1 and m1p1 revealed that the m^6^A binding site is located distantly from hnRNP A1’s canonical RNA-binding domain. Furthermore, several mRNAs bound by hnRNP A1 carry m^6^A modifications proximal to their binding sites (Figure S10 and Table S12), indicating that hnRNP proteins may recognize YAG sequences with adjacent m^6^A modifications. Our findings further suggest that proteins enriched in RNA pulldown assays interact with m^6^A-modified mRNAs, likely within the 3′-UTR region, and may play roles in post-transcriptional regulation, thereby influencing the fate of individual mRNAs, either independently or in conjunction with other proteins. These enriched proteins, containing RRM and KH domains, are involved in RNA splicing and pre-mRNA processing. With the high abundance of m^6^A modifications near splice sites, it is likely that m^6^A modifications can either recruit splice factors to specific splice sites or sequester splice sites to inhibit binding of the splice factors, thus promoting alternative splicing and splicing efficiency [61].

## 5. Conclusions

In conclusion, the contrasting preference of peptide sequences to m^6^A-modified and unmodified sequences demonstrates the ability of m^6^A modifications to influence the mRNA interactome. The identification of a specific peptide motif that recognizes m^6^A modifications located within various non-base-paired DRACH sequences indicates a shared mechanism of m^6^A recognition among these types of RNAs. However, proteins can employ contrasting mechanisms to recognize m^6^A modifications in regions of lower accessibility. The sequence within the hnRNP A1 RRM domain that aligns with the m^6^A-binding peptide, m1p1, is found to be crucial for the recognition of m^6^A-modified unstructured RNAs, suggesting the importance of the m1p1 peptide sequence in identifying m^6^A-binding proteins. Furthermore, these signature peptides hold significant promise for advancing theranostic strategies across various cancers, paving the way for more targeted and effective treatments.

## Data Availability

The data supporting the findings of this study are provided within the article and its Supplementary Materials. Raw data can be obtained from the corresponding author upon request. Furthermore, the mass spectrometry proteomics data are publicly available in the PRIDE, ProteomeXchange repository (dataset identifier PXD068096). All the raw data will be available upon request from the corresponding author.

## Supplementary Materials

The following supporting information can be downloaded at https://www.mdpi.com/article/10.3390/biom16010097/s1, Figure S1. Cartoon representation of a phage display biopanning cycle. Figure S2. Purified proteins were analyzed with an SDS-PAGE. Lanes 1, 2, 3, and 4 represent the full-length hnRNP A1, RBD of hnRNP A1, hnRNP A1-RBD single mutant, and hnRNP A1-RBD double mutants, respectively. Figure S3. Peptide sequences enriched in phage display against a m^6^A-modified MR3 RNA (CGCGCAACUm^6^AUG) are shown. The m^6^A methylation in MR3 is present within a non-DRACH (CUm^6^AUG) sequence. Figure S4. The Circular Dichroism of the m1p1 in (50 mM KCl, 20 mM sodium cacodylate, 0.5 mM EDTA, pH 7.6) is shown. Figure S5. Thermograms for the titration of MR1 RNA onto m1p1 and its mutant peptides are shown. Figure S6. Multiple sequence alignment of the YTH domains from 5 proteins with m1p1 peptide, using the ClustalW algorithm. The amino acid residues involved in the recognition of m^6^A methylation in proteins YTHDC1 (teal arrows) and YTHDF3 (plum arrows) are traced to the sequence of the respective protein. Figure S7. Subcellular localization from GO analysis of the 10 RNA-binding proteins enriched in the RNA pulldown assay. All 10 RNA-binding proteins were observed in the nucleus. Figure S8. Multiple sequence alignment of the RRM domains from 5 proteins enriched in RNA pulldown assay against m^6^A-modified RNA, using the ClustalW algorithm. Figure S9. Triplicates of thermograms from calorimetric titrations of hnRNP A1 RBD onto unmethylated RNA UR1 are shown. Figure S10. The number of nucleotides from the known hnRNP A1 binding site to the nearest m^6^A in the human genome is shown. Table S1. Table of oligonucleotides used for phage display and mutation with their molar extinction coefficients. Table S2. Washing conditions used in respective biopanning cycles. Table S3. Sequences and physicochemical properties of peptides enriched against MR1 RNA. The physicochemical properties of all the peptides, including theoretical molar extinction coefficient pI, and grand average of hydropathicity (GRAVY), were computed using the ProtParam tool of the ExPASy server (https://web.expasy.org/protparam/, accessed on 1 December 2025). Table S4. Sequences and physicochemical properties of peptides enriched against MR2 RNA. Table S5. Sequences and physicochemical properties of peptides enriched against UR1 RNA. Table S6. Sequences and physicochemical properties of peptides enriched against UR2 RNA. Table S7. Sequences and physicochemical properties of peptides enriched against MSR RNA. Table S8. Sequences and physicochemical properties of peptides enriched against MLR RNA. Table S9. Thermodynamic parameters for the binding of m1tp1 peptide and its mutants to m^6^A-modified RNA. Table S10. Comparison of proteins enriched in RNA pulldown assays against m^6^A-modified and unmodified RNAs. Rows of statistically significant protein hits are highlighted in light blue. RNA-binding proteins are shown in bold. Table S11. Thermodynamic parameters for the binding of hnRNP A1 RNA-binding domain and its mutants to m^6^A-modified RNA. Table S12. List of known genes with hnRNP A1 binding sites and m^6^A nucleotide modifications.
