# Supplementary material for: Identification of Key Sequence Motifs Essential for the Recognition of m6A Modification in RNA
Source: Biomolecules. 2026 Jan 7;16(1):97. doi: 10.3390/biom16010097 (PMC12838727; doi:10.3390/biom16010097)
Supplement: Supplementary file 1 [file biomolecules-16-00097-s001.zip › biomolecules-4019977-supplementary.pdf]

Supporting Information for

Proteins specifically recognize N6-methyladenosines in unstructured regions of RNAs through neighboring aromatic amino acid side chains.

Aftab U. Mollah[1], Rushdhi M. Rauff[1], Sudeshi M. Abedeera[1], Chathurani Ekanayake[1], Chamali P. K. Thalagaha Mudiyanselage[1], Minhchau To[1], Helen Piontkivska[2], and Sanjaya Abeysirigunawardena[1], *

1 Department of Chemistry and Biochemistry, Kent State University, Kent, Ohio, 44242, USA

2 Department of Biological Sciences, Kent State University, Kent, Ohio, 44242, USA

* To whom correspondence should be addressed. Tel: +1 (330) 672-2667; Fax: +1 (330) 672 3816; Email: sabeysir@kent.edu

This PDF file includes:

Figures S1 to S10 and the accompanying figure legends. Tables S1 to S12 and the accompanying table legends.

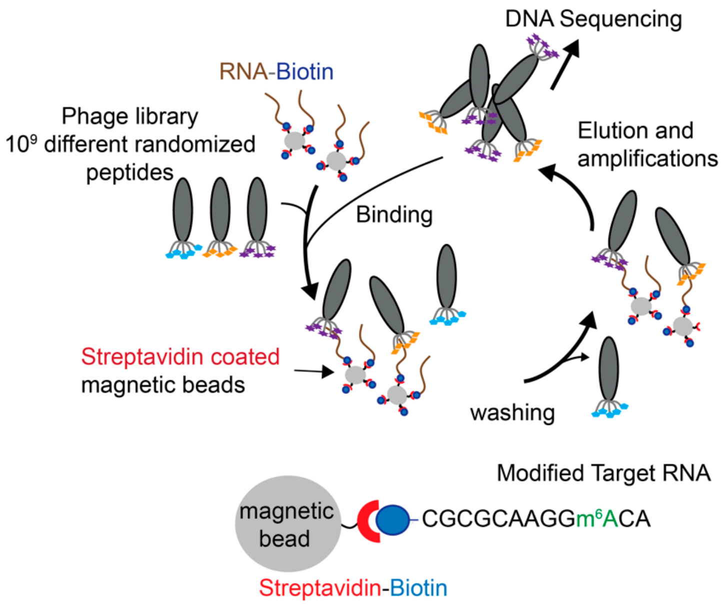

Figure S1. Cartoon representation of a phage display biopanning cycle.

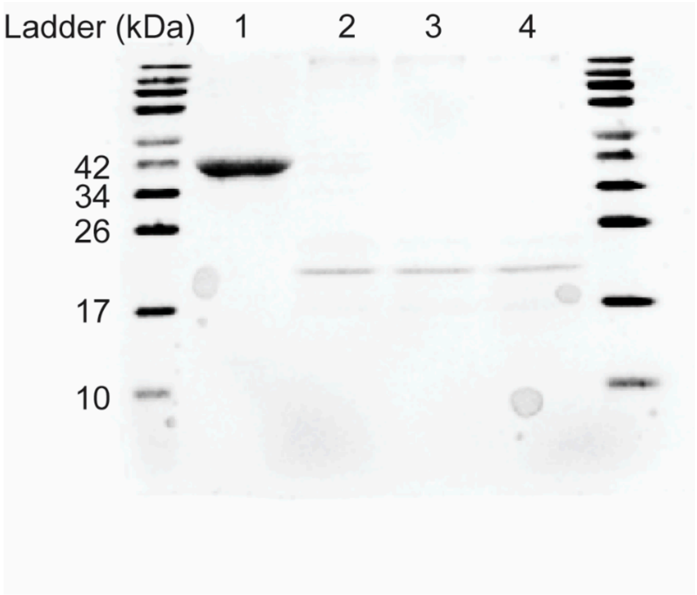

Figure S2. Purified proteins were analyzed with an SDS PAGE. Lanes 1, 2, 3, and 4 represent the full length hnRNP A1, RBD of hnRNP A1, hnRNP A1-RBD single mutant, and hnRNP A1-RBD double mutants, respectively.

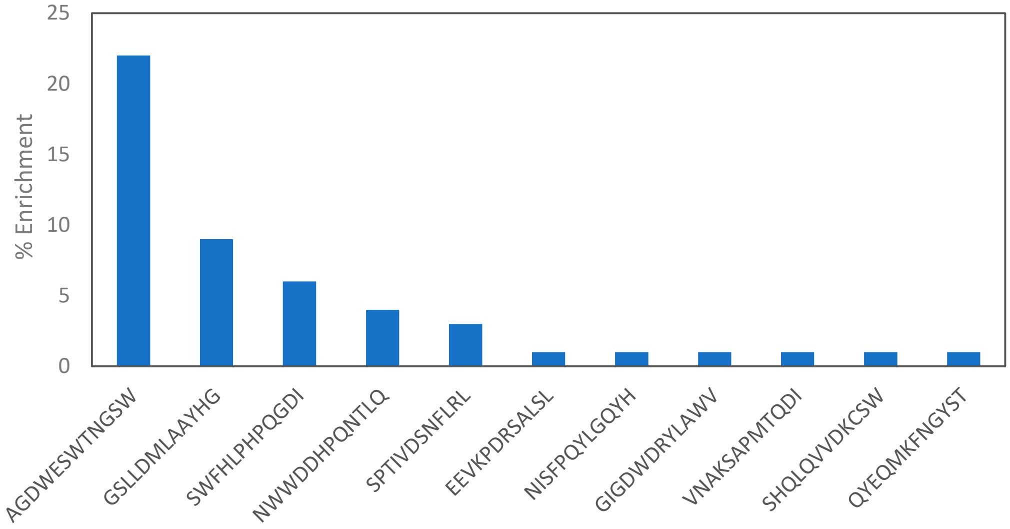

Figure S3. Peptide sequences enriched in phage display against a m^{6}A-modified MR3 RNA (CGCGCAACUm^{6}AUG) are shown. The m^{6}A methylation in MR3 is present within a non-DRACH (CUm^{6}AUG) sequence.

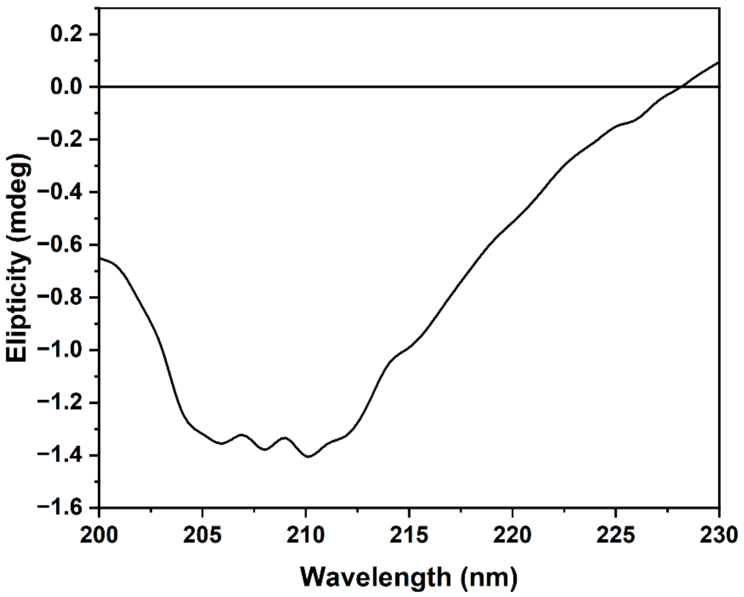

Figure S4. The Circular Dichroism of the m1p1 in (50 mM KCl, 20 mM Sodium cacodylate, 0.5 mM EDTA, pH 7.6) is shown.

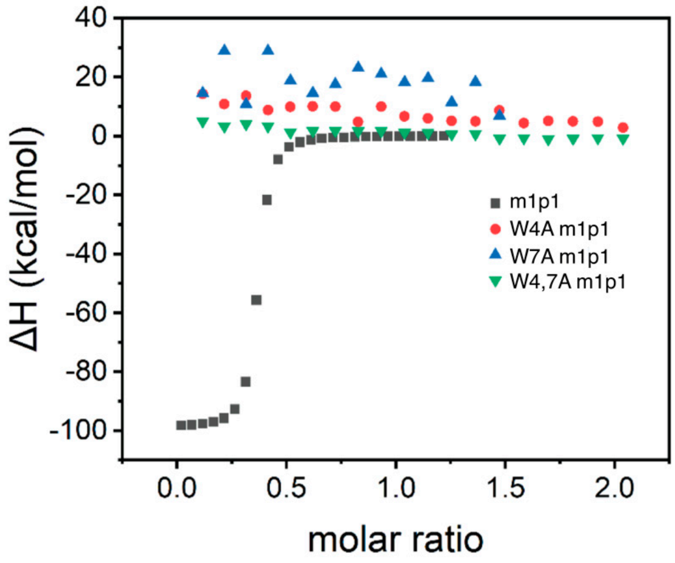

Figure S5. Thermograms for the titration of MR1 RNA onto m1p1 and its mutant peptides are shown.

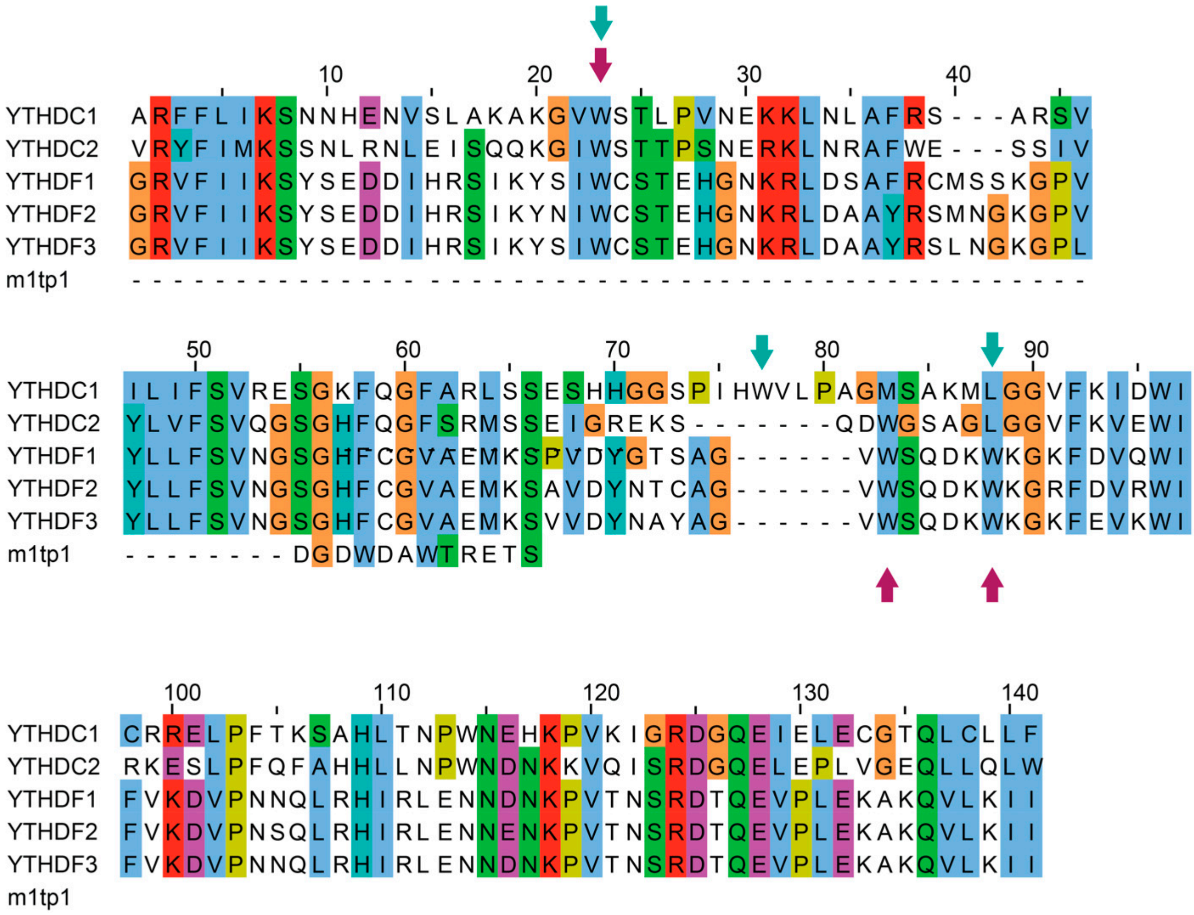

Figure S6. Multiple sequence alignment of the YTH domains from 5 proteins with m1p1 peptide, using the ClustalW algorithm. The amino acid residues involved in the recognition of m^{6}A methylation in proteins YTHDC1 (teal arrows) and YTHDF3 (plum arrows) are traced to the sequence of the respective protein.

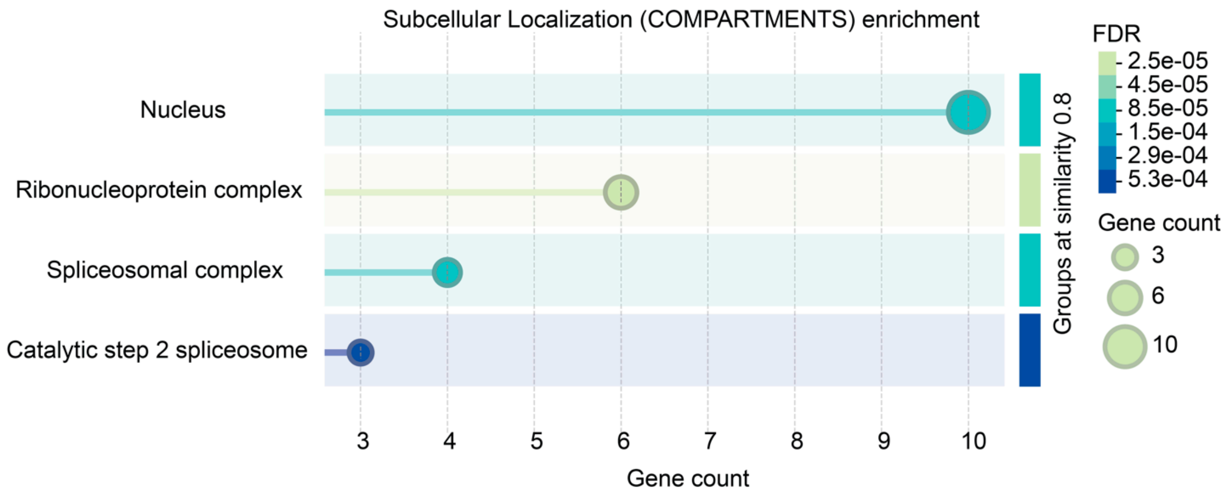

Figure S7. Subcellular localization from GO analysis of the 10 RNA-binding proteins enriched in the RNA pulldown assay. All 10 RNA-binding proteins were observed in the nucleus.

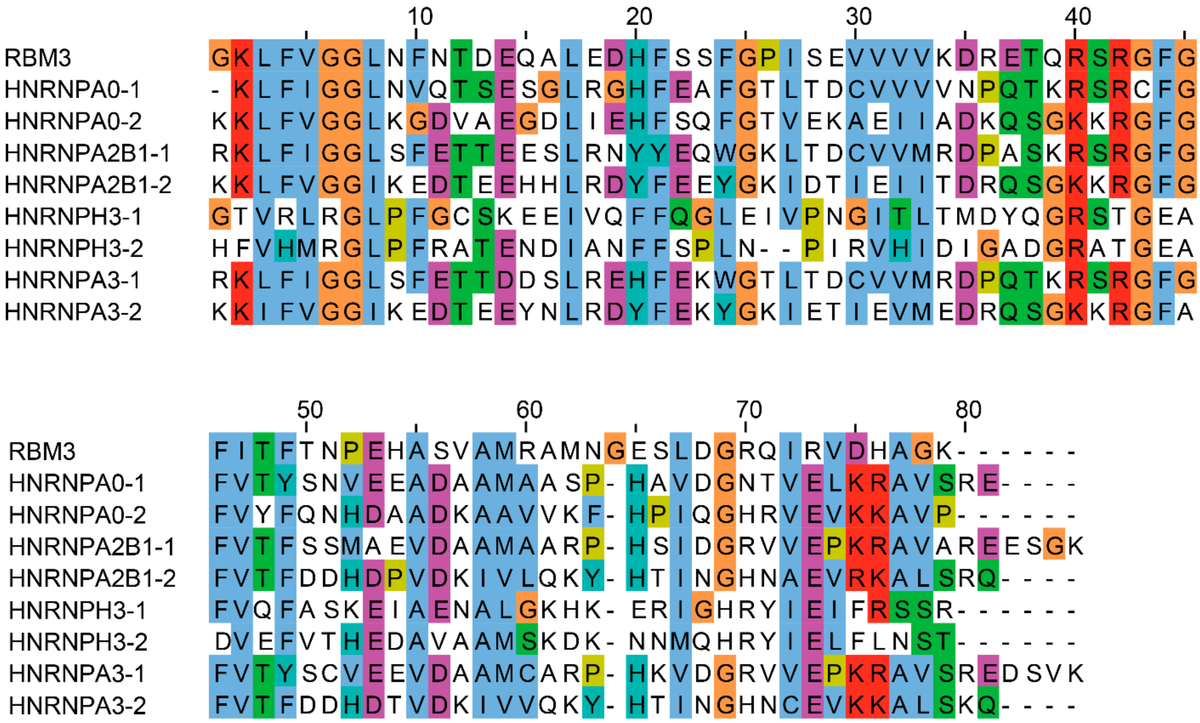

Figure S8. Multiple sequence alignment of the RRM domains from 5 proteins enriched in RNA pulldown assay against m^{6}A-modified RNA, using the ClustalW algorithm.

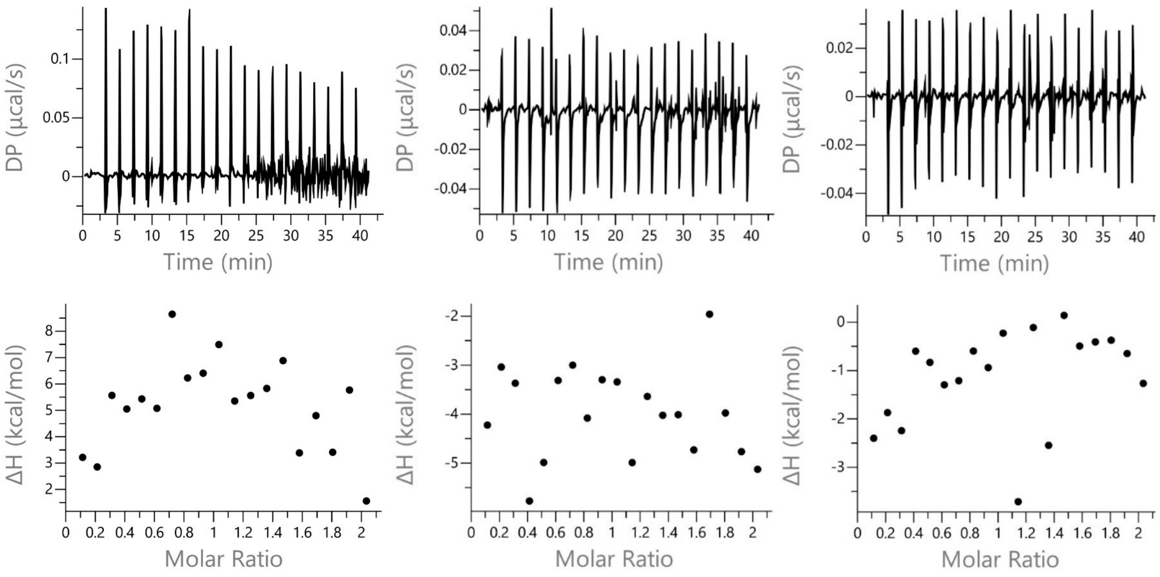

Figure S9. Triplicates of thermograms from calorimetric titrations of hnRNP A1 RBD onto unmethylated RNA UR1 are shown.

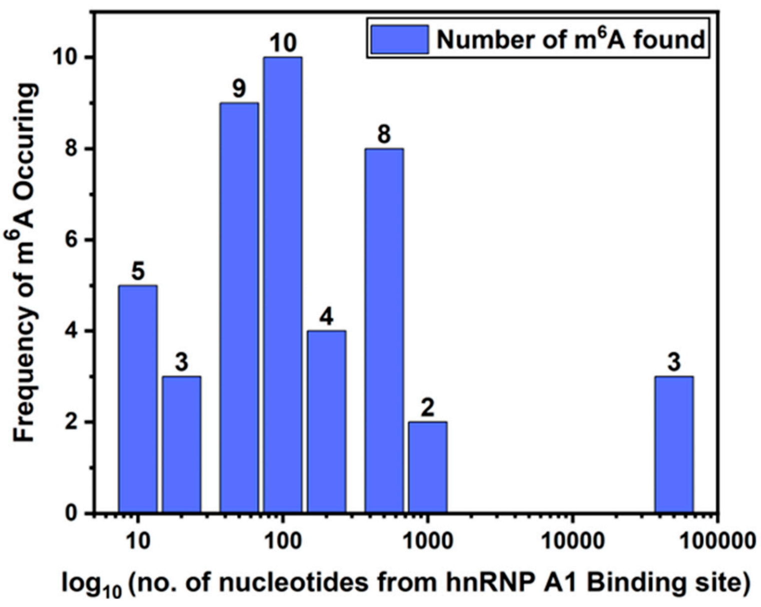

Figure S10. The number of nucleotides from the known hnRNP A1 binding site to the nearest m^{6}A in the human genome is shown.

Table S1. Table of oligonucleotides used for phage display and mutation with their molar extinction coefficients.

Oligo Name	Sequence (5'-3')	Extinction coefficient at 260 nm ($M^{-1}cm^{-1}$)
MR1	CGCGCAAGGm^{6}ACA	118,800
UR1	CGCGCAAGGACA	118,800
MR2	CGCGCAAAAm^{6}ACU	116,600
UR2	CGCGCAAAAACU	116,600
MSR	UGCGGm^{6}ACAACACACACUCAUUGUCCGC	254,500
MLR	UGACGCAAUCACGGm^{6}ACACAGUUGCGUC	262,700
MR3	CGCGCAACUm^{6}AUG	126,000
C-RNA	CGCGCAA	54,000
CS-RNA	UGACGCAAUCACACACUCAGUUGCGUC	246,500
CL-RNA	UGACGCAAUUACGGUUGCGUC	190,800

Table S2. Washing conditions used in respective biopanning cycles.

Conditions	Biopanning cycle			
	1	2	3	4
Target RNA (nM)	50	50	50	50
Tween-20	0.1%	0.2%	0.3%	0.3%
# of washes (1 mL/wash)	2	4	6	6
Competitor: tRNA			50 μg/ml	
Competitor: C-RNA (for MR1-3, and UR1-2 RNAs)				50 nM
Competitor: CS-RNA (for MSR RNA)				50 nM
Competitor: CL-RNA (for MLR RNA)				50 nM

Table S3. Sequences and physicochemical properties of peptides enriched against MR1 RNA. The physicochemical properties of all the peptides, including theoretical molar extinction coefficient pI, and grand average of hydropathicity (GRAVY), were computed using the ProtParam tool of the ExPASy server (https://web.expasy.org/protparam/).

Peptide name	Peptide Sequence	% Enrichment	Molar extinction coefficient ($M^{-1}cm^{-1}$)	GRAVY	pI
m1p1	DGDWDAWTRETS	18.75%	11000	-1.76	3.84
m1p2	AGDWMAYLAAMH	14.58%	6990	0.46	5.08
m1p3	DGDWDARTRETS	6.25%	5500	-2.06	4.23
m1p4	DGEWDAWTHETS	6.25%	11000	-1.65	3.91
m1p5	AVWHDNHPQFDT	4.17%	5500	-1.233	5.05
m1p6	DGEWDARTGETS	4.17%	5500	-1.717	3.92
m1p7	DGEWDAWTRETS	4.17%	11000	-1.758	3.92
m1p8	DGEWDARTHETS	4.17%	5500	-1.95	4.31
m1p9	DGEWDARTDERS	2.08%	5500	-2.292	4.11
m1p10	DGDWDAWTHETS	2.08%	11000	-1.65	3.84
m1p11	DGEWDARTDETS	2.08%	5500	-1.975	3.77
m1p12	DGEWDARRGEIS	2.08%	5500	-1.6	4.32
m1p13	DGEWDARTRETS	2.08%	5500	-2.058	4.32
m1p14	DGEWDARTGEIS	2.08%	5500	-1.283	3.92
m1p15	DGDWEAKTDEIS	2.08%	5500	-1.492	3.77
m1p16	DGEWDAWTGEIS	2.08%	11000	-0.983	3.43
m1p17	DREWDARTGEIS	2.08%	5500	-1.625	4.32
m1p18	DGEWDARTNERS	2.08%	5500	-2.292	4.32
m1p19	DGEWDARTHEIS	2.08%	5500	-1.517	4.31
m1p20	DGDWDARGRETS	2.08%	5500	-2.033	4.23
m1p21	DGEWDARRGKIS	2.08%	5500	-1.633	6.12
m1p22	DREWDAQTDKIS	2.08%	5500	-1.833	4.23
m1tp23	DREWDARTRETS	2.08%	5500	-2.4	4.78
m1p24	DREWDARTHETS	2.08%	5500	-2.292	4.75
m1p25	DGEWDARTGKIS	2.08%	5500	-1.317	4.56
m1p26	DREWDAQRDKIS	2.08%	5500	-2.15	4.68

Table S4. Sequences and physicochemical properties of peptides enriched against MR2 RNA

Peptide name	Peptide Sequence	% Enrichment	Molar extinction coefficient ($M^{-1}cm^{-1}$)	GRAVY	pI
m2p1	DGDWDAWTRETS	70.45%	11000	-1.76	3.84
m2p2	FKQDAWEAVDIR	2.27%	5500	-0.683	4.56
m2p3	GLDTWFNAHQKY	2.27%	6990	-1.042	6.74
m2p4	EGEGDAWTGENT	2.27%	5500	-1.6	3.5
m2p5	DADRYEYTNAII	2.27%	2980	-0.767	4.03
m2p6	DGDMVNWTRGIS	2.27%	5500	-0.633	4.21
m2p7	DKQDAWEARDIR	2.27%	5500	-1.933	4.68
m2p8	EGDGDARTRENS	2.27%	0	-2.25	4.32
m2p9	DGDWDARTRENS	2.27%	5500	-2.292	4.23
m2p10	NDDLNWYKTAIN	2.27%	6990	-1.183	4.21
m2p11	EGDWEANTPESC	2.27%	5500	-1.467	3.5
m2p12	VPVPKKMSLDPT	2.27%	0	-0.292	8.56
m2p13	HGNWEAQTCETT	2.27%	5500	-1.358	4.51
m2p14	LINNVYGFHSNL	2.27%	1490	0.242	6.74

Table S5. Sequences and physicochemical properties of peptides enriched against UR1 RNA

Peptide name	Peptide Sequence	% Enrichment	Molar extinction coefficient ($M^{-1}cm^{-1}$)	GRAVY	pI
u1p1	SSPDATWFWTYY	50.00%	-0.658	3.80	13980
u1p2	VECLYCWHPQFW	7.14%	0.075	5.24	12615
u1p3	LSGSLISNSHPQ	7.14%	-0.275	6.74	0
u1p4	LPFTKNAYSSSK	3.57%	-0.742	9.7	1490
u1p5	LEGPLDTQQYWR	3.57%	-1.317	4.37	6990
u1p6	SWQAHPMFPWSM	3.57%	-0.408	6.46	11000
u1p7	AGCWDCWTGVGN	3.57%	0.025	3.8	11125
u1p8	ANFGDLFLNHPQ	3.57%	-0.35	5.08	0
u1p9	VLRMQLWLGAYY	3.57%	0.617	8.56	8480
u1p10	VWMPSSSSLMED	3.57%	-0.075	3.67	5500
u1p11	FAQSHHQKMTGL	3.57%	-0.742	8.76	0
u1tp12	SPIRAVPLAVPY	3.57%	0.742	8.46	1490
u1tp13	HVTLSTSPSHPQ	3.57%	-0.742	6.92	0

Table S6. Sequences and physicochemical properties of peptides enriched against UR2 RNA

Peptide name	Peptide Sequence	% Enrichment	Molar extinction coefficient ($M^{-1}cm^{-1}$)	GRAVY	pI
u2p1	APTTWFNSDSIT	31.82%	5500	-0.342	3.8
u2p2	ARNTWFNSDSIT	29.55%	5500	-0.817	5.88
u2p3	APNTWFNSDSIT	4.55%	5500	-0.575	3.8
u2p4	ARDTWFYSDSII	2.27%	6990	-0.2	4.21
u2p5	APSTWFNNDSIT	2.27%	5500	-0.575	3.8
u2p6	AHTTWFNSDPID	2.27%	5500	-0.775	4.2
u2p7	APSTWFNSDSIT	2.27%	5500	-0.35	3.8
u2p8	AHNTWVKTEKIT	2.27%	5500	-0.875	8.64
u2p9	ELTTRFNSDSST	2.27%	0	-1.075	4.37
u2p10	ARNTGFNRDSIT	2.27%	0	-1.083	9.64
u2p11	ASIGRFNSDGIQ	2.27%	0	-0.317	5.88
u2p12	APTTAIHSEVPI	2.27%	0	0.392	5.24
u2p13	ARTTWFNSDSIT	2.27%	5500	-0.583	5.88
u2p14	APTTWIHFEVPI	2.27%	5500	0.467	5.24
u2p15	ARDTWFNSDSIT	2.27%	5500	-0.817	4.21
u2p16	APDSWFRTDAIT	2.27%	5500	-0.442	4.21
u2p17	ALIPWFNIDSIT	2.27%	5500	0.908	3.8
u2p18	APITWFNSDSIT	2.27%	5500	0.092	3.8

Table S7. Sequences and physicochemical properties of peptides enriched against MSR RNA

Peptide name	Peptide Sequence	% Enrichment	Molar extinction coefficient ($M^{-1}cm^{-1}$)	GRAVY	pI
msp1	GSLLDMLAAYHG	45.00%	1490	0.608	5.08
msp2	LVSNEYLLAHPQ	7.50%	1490	0	5.24
msp3	ILWHPQGDLPSG	5.00%	5500	-0.317	5.08
msp4	APMLPEWLGIFH	2.50%	5500	0.617	5.24
msp5	ASTPLYIREPHW	2.50%	6990	-0.667	6.79
msp6	YFDHRTHFLSVK	2.50%	1490	-0.625	8.6
msp7	VSVPGIITGTLR	2.50%	0	1.008	9.72
msp8	KLQFDDQQVWRI	2.50%	5500	-0.958	5.96
msp9	MSRLILIFXSVG	2.50%	0	1.583	9.5
msp10	SSTAPGLKLYPG	2.50%	1490	-0.175	8.31
msp11	HFLPLPGHMLHT	2.50%	0	0.183	7.02
msp12	VVGRAMAYSTIP	2.50%	1490	0.758	8.72
msp13	TFNNFYEARTEL	2.50%	1490	-0.833	4.53
msp14	AGDWESWTNGSW	2.50%	16500	-1.208	3.67
msp15	CFAGTPSILMLA	2.50%	0	1.617	5.52
msp16	AHLNRMTPGKVT	2.50%	0	-0.567	11
msp17	SVHHAMLPVSYP	2.50%	1490	0.283	6.66
msp18	LVSNEYLLAHPQ	2.50%	1490	0	5.24
msp19	DAHARALWRLSP	2.50%	5500	-0.5	9.61
msp20	NISFPQYLGQYH	2.50%	2980	-0.667	6.74

Table S8. Sequences and physicochemical properties of peptides enriched against MLR RNA

Peptide name	Peptide Sequence	% Enrichment	Molar extinction coefficient ($M^{-1}cm^{-1}$)	GRAVY	pI
mlp1	DGDWDAWTRETS	85.42%	11000	-1.758	3.84
mlp2	QSQSSDAVYSTN	2.08%	1490	-1.1	3.8
mlp3	GNSIAWLQDWRL	2.08%	11000	-0.342	5.84
mlp4	MDGRPHIKYNYR	2.08%	2980	-1.775	9.69
mlp5	HTASENWWYPRT	2.08%	12490	-1.65	6.75
mlp6	TFNQNSKTLPGF	2.08%	0	-0.767	8.41
mlp7	AYLEDWRSMSTR	2.08%	6990	-1.083	6.11
mlp8	SYTHLLHRSLPG	2.08%	1490	-0.425	8.51

Table S9. Thermodynamic parameters for the binding of m1tp1 peptide and its mutants to m^{6}A-modified RNA

RNA	Peptide	Kd (nM)	ΔH (kcal/mol)	ΔG (kcal/mol)	TΔS (kcal/mol)
MR1	m1p1	2.8 ± 0.7	-98 ± 18	-12.3 ± 0.1	86 ± 18
	AW m1p1	N.D.	N.D.	N.D.	N.D.
	WA m1p1	N.D.	N.D.	N.D.	N.D.
	AA m1p1	N.D.	N.D.	N.D.	N.D.
MR2	m1p1	6.2 ± 0.3	-28 ± 1	-11.63 ± 0.05	17 ± 1
MLR	m1p1	34 ± 8	-29 ± 3	-11 ± 0.1	18 ± 3
MSR	m1p1	125 ± 28	-14.3 ± 0.6	-9.8 ± 0.1	4.5 ± 0.8
UR1	m1p1	N.D.	N.D.	N.D.	N.D.

Table S10. Comparison of proteins enriched in RNA pulldown assays against m[6]A-modified and unmodified RNAs. Rows of statistically significant protein hits are highlighted in light blue. RNA-binding proteins are shown in bold.

Protein	Accession	Gene ID	PSM	LFQ ratio M/U	p-value
Lysozyme C	P61626	LYZ	9	0	0.002222
Hemoglobin subunit beta	P68871	HBB	9	0	0.373901
Hemoglobin subunit alpha	P69905	HBA1	4	0	0.373901
Endoplasmic reticulum chaperone BiP	P11021	HSPA5	14	0	0.373901
Calmodulin-like protein 5	Q9NZT1	CALML5	30	0.270832	0.011237
Serpin B3	P29508	SERPINB3	3	0.33273	0.340514
ATP-dependent RNA helicase A	Q08211	DHX9	3	0.334751	0.360917
60S ribosomal protein L23	P62829	RPL23	2	0.353956	0.417807
Pyruvate kinase PKM	P14618	PKM	15	0.377481	0.008912
Serum albumin	P02768	ALB	15	0.534078	0.214902
40S ribosomal protein S4, X isoform	P62701	RPS4X	13	0.600407	0.15795
Protein S100-A9	P06702	S100A9	18	0.604052	0.236465
DNA topoisomerase 1	P11387	TOP1	12	0.612481	0.199521
Dermcidin	P81605	DCD	23	0.625884	0.193635
60S ribosomal protein L13	P26373	RPL13	2	0.672442	0.353941
Prolactin-inducible protein	P12273	PIP	9	0.721315	0.446386
Pre-mRNA-splicing factor SPF27	O75934	BCAS2	20	0.791009	0.020454
60S ribosomal protein L27a	P46776	RPL27A	3	0.798665	0.32013
Catalase	P04040	CAT	5	0.807493	0.87615
40S ribosomal protein S11	P62280	RPS11	11	0.859107	0.669315
60S ribosomal protein L26	P61254	RPL26	10	0.878498	0.500101
Histone H1.3	P16402	HIST1H1D	39	0.884772	0.577914
40S ribosomal protein S6	P62753	RPS6	25	0.888196	0.475642
Heat shock 70 kDa protein 1-like	P34931	HSPA1L	11	0.898146	0.840858
60 kDa heat shock protein, mitochondrial	P10809	HSPD1	6	0.919649	0.935747
40S ribosomal protein S7	P62081	RPS7	10	0.939982	0.694116
Ubiquitin-40S ribosomal protein S27a	P62979	RPS27A	22	0.960255	0.872398
Leukotriene A-4 hydrolase	P09960	LTA4H	101	0.971918	0.685354
Probable ATP-dependent RNA helicase DDX17	Q92841	DDX17	16	0.990848	0.964775
Splicing factor, proline- and glutamine-rich	P23246	SFPQ	7	1.009104	0.984017
Probable ATP-dependent RNA helicase DDX5	P17844	DDX5	22	1.015941	0.926279
Histone H2B type 2-E	Q16778	HIST2H2BE	34	1.029538	0.796927
60S ribosomal protein L23a	P62750	RPL23A	41	1.048339	0.64158
Desmocollin-1	Q08554	DSC1	4	1.058646	0.96251
Peptidyl-prolyl cis-trans isomerase A	P62937	PPIA	17	1.074795	0.889124
Tropomyosin alpha-3 chain	P06753	TPM3	297	1.077021	0.551798

Protein	Accession	Gene ID	PSM	LFQ ratio M/U	p-value
40S ribosomal protein S17	P08708	RPS17	61	1.083375	0.627338
Histone H3.3	P84243	H3F3A	9	1.1012	0.135977
Peroxiredoxin-1	Q06830	PRDX1	8	1.111431	0.610882
Junction plakoglobin	P14923	JUP	26	1.12894	0.881985
Tropomodulin-3	Q9NYL9	TMOD3	20	1.132289	0.702722
Treacle protein	Q13428	TCOF1	12	1.13313	0.477986
Myosin light polypeptide 6	P60660	MYL6	316	1.133321	0.209495
Tubulin beta-4B chain	P68371	TUBB4B	64	1.143939	0.203536
Profilin-1	P07737	PFN1	25	1.144634	0.377479
Tropomyosin alpha-1 chain	P09493	TPM1	271	1.146674	0.356935
Heat shock protein beta-1	P04792	HSPB1	15	1.151844	0.537674
S-phase kinase-associated protein 1	P63208	SKP1	24	1.158382	0.62179
Calmodulin-3	P0DP25	CALM3	13	1.172239	0.21328
14-3-3 protein zeta/delta	P63104	YWHAZ	11	1.18951	0.109655
Corneodesmosin	Q15517	CDSN	3	1.201004	0.884887
40S ribosomal protein S8	P62241	RPS8	31	1.205988	0.475832
Desmoplakin	P15924	DSP	42	1.209597	0.819876
Heterogeneous nuclear ribonucleoprotein H2	P55795	HNRNPH2	17	1.222233	0.574666
Protein dpy-30 homolog	Q9C005	DPY30	11	1.228696	0.768402
Elongation factor 1-alpha 1	P68104	EEF1A1	42	1.231455	0.62819
Tropomyosin alpha-4 chain	P67936	TPM4	351	1.245154	0.135093
Myosin-9	P35579	MYH9	187	1.271741	0.066014
Histone-binding protein RBBP4	Q09028	RBBP4	6	1.272441	0.352339
60S acidic ribosomal protein P1	P05386	RPLP1	10	1.274046	0.120032
Chromatin complexes subunit BAP18	Q8IXM2	BAP18	15	1.282074	0.08769
Calumenin	O43852	CALU	10	1.289866	0.056577
Tubulin alpha-1B chain	P68363	TUBA1B	56	1.306786	0.257487
Tubulin beta chain	P07437	TUBB	73	1.324283	0.067649
Cytoskeleton-associated protein 4	Q07065	CKAP4	12	1.367869	0.169062
Leucine-rich repeat flightless-interacting protein 2	Q9Y608	LRRFIP2	18	1.393321	0.120702
Src substrate cortactin	Q14247	CTTN	22	1.396599	0.039709
40S ribosomal protein S12	P25398	RPS12	19	1.42486	0.073944
Chromobox protein homolog 3	Q13185	CBX3	7	1.453565	0.101984
Myosin regulatory light polypeptide 9	P24844	MYL9	18	1.468244	0.053449
Fructose-bisphosphate aldolase A	P04075	ALDOA	14	1.481942	0.452718
Myosin-10	P35580	MYH10	64	1.504204	0.016852
Stathmin	P16949	STMN1	11	1.523099	0.014269
40S ribosomal protein S15a	P62244	RPS15A	9	1.524519	0.15549
60S acidic ribosomal protein P2	P05387	RPLP2	21	1.532928	0.167218

Protein	Accession	Gene ID	PSM	LFQ ratio M/U	p-value
14-3-3 protein beta/alpha	P31946	YWHAB	6	1.538141	0.358544
Myosin regulatory light chain 12B	O14950	MYL12B	72	1.564963	0.053638
Tubulin beta-6 chain	Q9BUF5	TUBB6	14	1.574606	0.77342
Phosphoglycerate kinase 1	P00558	PGK1	6	1.596959	0.596078
40S ribosomal protein S21	P63220	RPS21	18	1.606422	0.002076
ATP synthase subunit d, mitochondrial	O75947	ATP5PD	6	1.656917	0.029554
Myosin light chain 6B	P14649	MYL6B	62	1.700652	0.085253
Signal recognition particle 9 kDa protein	P49458	SRP9	14	1.713942	0.008186
Heterogeneous nuclear ribonucleoprotein A1-like 2	Q32P51	HNRNPA1L2	19	1.791109	0.32494
Nascent polypeptide-associated complex subunit alpha	Q13765	NACA	12	1.933686	0.034479
Elongation factor 1-beta	P24534	EEF1B2	12	1.96194	0.001631
Heterogeneous nuclear ribonucleoprotein M	P52272	HNRNPM	9	2.005809	0.156951
Histone H2A type 2-C	Q16777	HIST2H2AC	43	2.024561	0.097544
RNA-binding protein 3	P98179	RBM3	4	2.040128	0.047
Annexin A2	P07355	ANXA2	31	2.043472	0.549972
Alpha-enolase	P06733	ENO1	36	2.080886	0.244975
Glyceraldehyde-3-phosphate dehydrogenase	P04406	GAPDH	30	2.195244	0.234668
Desmoglein-1	Q02413	DSG1	80	2.26464	0.450165
Heat shock protein HSP 90-beta	P08238	HSP90AB1	41	2.296884	0.155426
Nucleolin	P19338	NCL	15	2.31098	0.198923
Far upstream element-binding protein 2	Q92945	KHSRP	22	2.324192	0.005707
Desmocollin-3	Q14574	DSC3	5	2.364341	0.501188
Heterogeneous nuclear ribonucleoproteins C1/C2	P07910	HNRNPC	21	2.389726	0.268516
Histone H4	P62805	HIST1H4A	73	2.441378	0.109367
Elongation factor 1-delta	P29692	EEF1D	14	2.559664	0.00082
Heterogeneous nuclear ribonucleoprotein H	P31943	HNRNPH1	331	2.692775	0.055061
Apolipoprotein D	P05090	APOD	2	2.798096	0.083742
Heat shock protein HSP 90-alpha	P07900	HSP90AA1	30	2.948773	0.348374
Cofilin-1	P23528	CFL1	8	3.15161	0.060936
Heat shock cognate 71 kDa protein	P11142	HSPA8	25	3.252472	0.295954
40S ribosomal protein S23	P62266	RPS23	24	3.344734	0.00794
Nucleophosmin	P06748	NPM1	40	3.369059	0.007108
Actin, cytoplasmic 1	P60709	ACTB	298	3.373785	0.023105
Heterogeneous nuclear ribonucleoprotein H3	P31942	HNRNPH3	39	3.388072	0.009237
A-kinase anchor protein 2	Q9Y2D5	AKAP2	5	4.073276	0.05149
Apolipoprotein F	Q13790	APOF	33	4.36054	0.377728
F-actin-capping protein subunit alpha-1	P52907	CAPZA1	10	4.574651	0.086999
Caspase-14	P31944	CASP14	17	4.887646	0.423903

Protein	Accession	Gene ID	PSM	LFQ ratio M/U	p-value
Heterogeneous nuclear ribonucleoprotein K	P61978	HNRNPK	17	4.917925	0.107596
Heterogeneous nuclear ribonucleoprotein F	P52597	HNRNPF	22	5.487533	0.154658
Heterogeneous nuclear ribonucleoprotein U	Q00839	HNRNPU	121	5.5306	0.002242
40S ribosomal protein S3a	P61247	RPS3A	12	5.904176	0.054539
Receptor of activated protein C kinase 1	P63244	RACK1	4	6.507873	0.242543
Heterogeneous nuclear ribonucleoprotein A3	P51991	HNRNPA3	26	6.82709	0.028942
Heterogeneous nuclear ribonucleoproteins A2/B1	P22626	HNRNPA2B1	88	11.91875	0.014285
Zinc-alpha-2-glycoprotein	P25311	AZGP1	6	13.27194	0.408688
60S ribosomal protein L17	P18621	RPL17	12	16.23808	0.087827
Histone H2A.V	Q71UI9	H2AFV	20	26.14249	0.029201
Drebrin	Q16643	DBN1	8	M only	1.06E-07
Far upstream element-binding protein 1	Q96AE4	FUBP1	3	M only	0.001045
Heterogeneous nuclear ribonucleoprotein A0	Q13151	HNRNPA0	8	M only	0.024425
ADP/ATP translocase 3	P12236	SLC25A6	10	M only	0.040858
POTE ankyrin domain family member J	P0CG39	POTEJ	101	M only	0.042994
Mitochondrial import receptor subunit TOM22 homolog	Q9NS69	TOMM22	9	M only	0.080246
Actin, alpha skeletal muscle	P68133	ACTA1	147	M only	0.116298
Voltage-dependent anion-selective channel protein 2	P45880	VDAC2	3	M only	0.129512
Chromobox protein homolog 1	P83916	CBX1	8	M only	0.155011
Malate dehydrogenase, mitochondrial	P40926	MDH2	3	M only	0.166401
60S ribosomal protein L5	P46777	RPL5	6	M only	0.177782
Matrin-3	P43243	MATR3	3	M only	0.178352
ATP synthase subunit alpha, mitochondrial	P25705	ATP5F1A	5	M only	0.197276
Cathepsin D	P07339	CTSD	10	M only	0.373901
Gamma-glutamylcyclotransferase	O75223	GGCT	4	M only	0.373901
Hemoglobin subunit zeta	P02008	HBZ	3	M only	0.373901
Creatine kinase B-type	P12277	CKB	5	M only	0.373901
Protein-glutamine gamma-glutamyltransferase E	Q08188	TGM3	6	M only	0.373901
POTE ankyrin domain family member F	A5A3E0	POTEF	114	NQ	NQ
Tubulin alpha-1C chain	Q9BQE3	TUBA1C	41	NQ	NQ
U6 snRNA-associated Sm-like protein LSm3	P62310	LSM3	6	NQ	NQ
Beta-actin-like protein 2	Q562R1	ACTBL2	20	NQ	NQ
U6 snRNA-associated Sm-like protein LSm1	O15116	LSM1	7	NQ	NQ
Fatty acid-binding protein 5	Q01469	FABP5	7	NQ	NQ
Myosin light chain 3	P08590	MYL3	8	NQ	NQ
U6 snRNA-associated Sm-like protein LSm5	Q9Y4Y9	LSM5	3	NQ	NQ
Protein S100-A8	P05109	S100A8	3	NQ	NQ
Protein S100-A7	P31151	S100A7	3	NQ	NQ

Protein	Accession	Gene ID	PSM	LFQ ratio M/U	p-value
Secreted Ly-6/uPAR-related protein 1	P55000	SLURP1	3	NQ	NQ
Eukaryotic translation initiation factor 6	P56537	EIF6	4	NQ	NQ
Annexin A1	P04083	ANXA1	4	NQ	NQ
Protein quaking	Q96PU8	QKI	3	NQ	NQ
Kallikrein-7	P49862	KLK7	2	NQ	NQ
Protein disulfide-isomerase	P07237	P4HB	4	NQ	NQ
Histone-binding protein RBBP7	Q16576	RBBP7	2	NQ	NQ
RNA-binding protein 14	Q96PK6	RBM14	3	NQ	NQ
Elongation factor 2	P13639	EEF2	3	NQ	NQ
Plakophilin-1	Q13835	PKP1	2	NQ	NQ

Table S11 Thermodynamic parameters for the binding of hnRNP A1 RNA-binding domain and its mutants to m^{6}A-modified RNA

RNA	Peptide	K_d (μM)	ΔH (kcal/mol)	ΔG (kcal/mol)	TΔS (kcal/mol)
MTR1	wt-RBD	2.8 ± 0.7	-98 ± 17	-12.3 ± 0.1	86 ± 18
	AW RBD	N.D.	N.D.	N.D.	N.D.
	AA RBD	N.D.	N.D.	N.D.	N.D.

Table S12 List of known genes with hnRNP A1 binding sites and m^{6}A nucleotide modifications.

sl no.	Genes	Binding sites	Reference	Chromosome number	m^{6}A Distance on mRNA
1	APOL1	A consensus cis-acting element in exon 4	Cheatham et al. (2018)	22	159
2	APP	Alu element in introns 6 and 8	Donev et al. (2007)	21	13, 36, 60
3	AR	UAGGGA in splice sites	Nadiminty et al. (2015)	X	3
4	ATP7B	ESS sites in exon 12	Lin et al. (2015)	13	353
5	ATM	Alu - derived Intronic splicing enhancer (ISE) in intron 20	Pastor and Pagani, (2011)	11	9
6	Bcl-x	5′ splice site	Cloutier et al. (2018)	20	5, 12, 21, 573
7	beta-tropomyosin	G-rich intronic sequence (S3) downstream of exon 6B	Expert-Bezancon et al. (2004)	9	301, 543
8	CD44	Splice regulatory elements in exon v 5	Matter et al. (2000)	11	44
9	CDK2	GUAGUAGU in intron 4	Yu et al. (2015)	12	45, 67
10	CEACAM1	3′ to exon 7	Dery et al. (2011)	19	542, 480
11	Fas	GAGGAA at 5′ splice site of exon 5	Oh et al. (2013)	10	3837, 19782
12	INSR	AGGGA sites in intron 10	Talukdar et al. (2011)	19	33000
13	Mag	UAGGU at the 5′ splice site of Magexon 12	(Zhao et al.,2010; Zearfoss et al.,2013)	19	>5000
14	MYLK	UAGGGA in Intron 10	Mascarenhas et al. (2018)	3	450-1000
15	Max	intronic region in intron 4	Babic et al. (2013)	14	4
16	PKM	Intronic UAGGGC sequence flanking exon 9	David et al. (2010)	15	50, 69, 73, 93, 97
